# Potential implications of granzyme B in keloids and hypertrophic scars through extracellular matrix remodeling and latent TGF-β activation

**DOI:** 10.3389/fimmu.2024.1484462

**Published:** 2025-01-16

**Authors:** Alexandre Aubert, Jenna Goeres, Amy Liu, Martin Kao, Katlyn C. Richardson, Karen Jung, Boris Hinz, Richard I. Crawford, David J. Granville

**Affiliations:** ^1^ International Collaboration on Repair Discoveries (ICORD) Centre, Vancouver Coastal Health Research Institute (VCHRI), University of British Columbia (UBC), Vancouver, BC, Canada; ^2^ Department of Pathology and Laboratory Medicine, University of British Columbia (UBC), Vancouver, BC, Canada; ^3^ British Columbia Professional Firefighters’ Burn and Wound Healing Group, Vancouver Coastal Health Research Institute (VCHRI), Vancouver, BC, Canada; ^4^ Laboratory of Tissue Repair and Regeneration, Keenan Research Institute for Biomedical Science of the St. Michael’s Hospital, Toronto, ON, Canada; ^5^ Faculty of Dentistry, University of Toronto, Toronto, ON, Canada; ^6^ Department of Dermatology and Skin Science, University of British Columbia (UBC), Vancouver, BC, Canada

**Keywords:** keloids, hypertrophic scars, granzymes, inflammation, extracellular matrix remodeling, TGF-β activation

## Abstract

Keloid scars (KS) and hypertrophic scars (HS) are fibroproliferative wound healing defects characterized by excessive accumulation of extracellular matrix (ECM) in the dermis of affected individuals. Although transforming growth factor (TGF)-β is known to be involved in the formation of KS and HS, the molecular mechanisms responsible for its activation remain unclear. In this study we investigated Granzyme B (GzmB), a serine protease with established roles in fibrosis and scarring through the cleavage of ECM proteins, as a potential new mediator of TGF-β activation in KS and HS. Increased GzmB-positive mast cells were identified in the dermis of KS and HS but not healthy skin controls. Elevated levels of substance P, a neuropeptide involved in mast cell degranulation, suggest that GzmB is released extracellularly, as confirmed by the significant reduction of the established extracellular GzmB substrate decorin in KS and HS. Similarly, presence of latent TGF-β binding protein 1 (LTBP1), a protein involved in the extracellular tethering of latent TGF-β, was disrupted proximal to the dermal-epidermal junction (DEJ) of GzmB^high^ KS and HS lesions. Using LTBP1-enriched medium as well as purified LTBP1, its cleavage by GzmB was confirmed *in vitro*. Increased TGF-β/Smad signaling pathway was observed in keratinocytes treated with GzmB-digested LTBP1 and was abolished by the addition of a pan-TGF-β inhibitor, suggesting that GzmB cleavage of LTBP1 contributes to TGF-β activation. In dermal fibroblasts, GzmB also cleaved cell-derived LTBP1 and induced TGF-β activation through the cleavage of one or more unidentified fibroblast-secreted proteins. Altogether, the present results suggest that GzmB contributes to KS and HS through ECM remodeling and TGF-β activation.

## Graphical Abstract

Graphical Abstract.

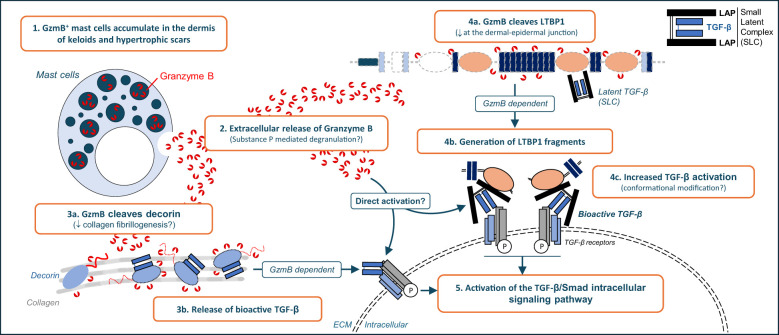

## Introduction

Keloid scars (KS) and hypertrophic scars (HS) represent two forms of pathologic scarring characterized by excessive extracellular matrix (ECM) accumulation, fibroblast proliferation, aberrant proteolysis, and secretion of pro-inflammatory mediators (1). Stemming from a connective tissue response to trauma, inflammation, surgery, or burns, KS and HS represent major aesthetic burdens for patients and are often associated with reduced quality of life due to mobility issues, pruritus, pain, and discomfort (1).

KS and HS share numerous hallmark features including chronic inflammation (2), infiltration of mast cells and macrophages (3, 4), as well as dermal fibrosis (5); however, they also exhibit distinct histological and underlying molecular attributes. KS are rounded protuberances that continuously grow beyond the confines of the wound margins and rarely regress over time, while HS are raised lesions that remain confined within the wound margins (1). In opposition to the increased type I to type III collagen ratio leading to the formation of abnormally thick collagen bundles in KS, HS are characterized by wavy type-III collagen rich bundles accumulating in the dermis (1). Clinical management options for KS and HS overlap and include surgery, occlusive dressing, compression therapies, and intralesional steroid administration (6). Nevertheless, efficacy of these approaches can vary between patients, with adverse side effects and potential recurrence, especially for KS. There is consequently an urgent need to better understand the pathobiology of KS and HS to develop more effective and safer therapies.

Another key molecular commonality between KS and HS is the up-regulated activity of transforming growth factor (TGF)-β compared to healthy skin (5). TGF-β exists in three isoforms in humans (β1, β2 and β3) and is produced by many cell types in an inactive form. Synthesized as a disulfide bonded dimeric precursor, TGF-β is proteolytically separated from its latency associated pro-peptide LAP by furin protein convertase during intracellular processing (7). Though cleaved, TGF-β and LAP remain non-covalently attached to form a small latent complex (SLC), where the LAP can act as a molecular *“straitjacket*” to prevent TGF-β from interacting with its cell surface receptors (8). While the SLC can be found in a soluble extracellular form, it is typically tethered to a presenting protein partner. In the ECM, the SLC is covalently bound to latent TGF-β binding protein-1 (LTBP1) to create a large latent complex (LLC) that is anchored to the fibronectin- and fibrillin-rich ECM (9). Bioactive TGF-β can also be sequestered into the skin dermis by interacting with decorin (10), a structural dermatan-sulfate proteoglycan involved in collagen fibrillogenesis (11).

Extracellular activation of TGF-β is a tightly regulated process that can be achieved through different mechanisms depending on the cell type involved. TGF-β can be activated independent of proteases through conformational modifications induced by mechanical stress (8, 12). In fact, active TGF-β is released by cell contractile forces applied to the SLC by integrins from the αv family, including αvβ6 expressed by epithelial cells (13), αvβ1 (14) and αvβ5 (15) expressed by fibroblastic cells, as well as αvβ8 predominantly expressed by immune cells (16). Likewise, TGF-β bioactive sites appear to be unmasked upon binding of the SLC to ECM molecules such as thrombospondin-1 (17), ADAMTS1 (18), and members of the tenascin family (19). Finally, soluble TGF-β can also be fully released from the SLC by proteolytic degradation of the LAP pro-peptide by extracellular proteases, including but not limited to plasmin (20), calpain (21), and matrix metalloproteinases (MMPs (22, 23)). Once activated, TGF-β can engage TGF-β receptors type I (TβRI, also known as ALK5) and type II (TβRII) at the cell surface to promote a canonical intracellular signaling pathway resulting in Smad2 and Smad3 phosphorylation (7). In cooperation with Smad4, phospho-Smad2/3 can translocate into the nucleus where they act as transcription factors to regulate the expression of TGF-β responsive genes involved in cytostasis and apoptosis (24), immuno-modulation (25), myofibroblast differentiation (26), as well as ECM protein synthesis (27).

TGF-β has been extensively investigated in the context of wound healing, fibrosis, and scarring (28) and is believed to play an important role in the pathogenesis of KS and HS (29). Recent characterization of the keloidal matrisome by mass spectrometry revealed significantly elevated levels of TGF-β1, as well as several of its extracellular activators, in the ECM of KS (30). A dysregulated TβRI/TβRII ratio (31), alongside increased Smad2 expression (32), is observed in fibroblasts derived from KS and HS which demonstrate a higher expression of αSMA, collagens and connective tissue growth factor (CTGF) after TGF-β stimulation compared to healthy fibroblasts (33–36). Alteration of the TGF-β signaling pathway is also possibly linked to the loss and/or reduction of decorin in KS (37, 38) and HS (39, 40). *In vitro*, the addition of decorin to HS fibroblasts inhibits their activation into scar-producing myofibroblasts by TGF-β (33), and decorin overexpression in keloidal fibroblasts reduces type I and type III collagen synthesis (37). Nevertheless, the molecular mechanisms involved in the regulation of TGF-β bioavailability in KS and HS remain to be fully elucidated.

Granzyme B (GzmB) is a serine protease with aspase-like activity extensively studied for its implications in immune cell-mediated apoptosis. Initially identified in the lytic granules of cytotoxic T lymphocytes (CTLs) and natural killer (NK) cells, GzmB is delivered into the cytoplasm of targeted cells during immunological synapse formation (41). Once internalized aided by the pore forming protein perforin, GzmB can promote both caspase-dependent and -independent apoptosis through the cleavage of specific intracellular substrates (41). Nevertheless, either passively released by CTLs (42, 43) or produced by non-immune cells as well as other immune cells without cytotoxic potential [reviewed in (44)], GzmB can be secreted and accumulates in the extracellular space in conditions characterized by dysregulated or excessive inflammation. Due to the lack of an endogenous extracellular inhibitor in humans (45, 46), GzmB retains its proteolytic activity outside of cells and can contribute to disease progression through multiple extracellular pathways [previously reviewed in (44)].

In contrast to its absence in healthy individuals, GzmB accumulates extracellularly in a wide range of dermatological conditions including atopic dermatitis (47), psoriasis (48), and autoimmune blistering diseases (49, 50). GzmB is also elevated in human pressure injuries (51) as well as in several animal models of metabolically (52) or diabetically impaired (53, 54) wound healing. In these contexts, GzmB can directly contribute to both scarring and/or delayed wound closure through the cleavage of specific ECM proteins – fibronectin and vitronectin (55), type IV (56) and type VII (49) collagens, laminin (55), and biglycan (57). GzmB also directly cleaves decorin, leading to impaired collagen fibrillogenesis, associated with the release and subsequent activation of TGF-β (54, 57). To our knowledge, the role of GzmB has never been evaluated in KS and HS.

To investigate the implication of GzmB in KS and HS we immunostained human skin samples and detected GzmB^+^ mast cells exhibiting degranulation profiles accumulating in the dermis of KS and HS but not healthy skin controls. Levels of decorin, a well characterized extracellular GzmB substrate, were significantly reduced in the dermis of KS and HS. We also identified the loss of LTBP1 at the dermal epidermal junction of GzmB^high^, but not GzmB^low^, lesions. *In vitro*, recombinant human LTBP1 was proteolytically cleaved by recombinant human GzmB. Increased activation of the TGF-β/Smad signaling pathway was also identified in cells treated with LTBP1 (conditioned medium or purified recombinant protein) pre-digested by GzmB. This GzmB/LTBP1-mediated Smad2 phosphorylation was abolished by a pan-TGF-β inhibitor, suggesting that GzmB cleavage of LTBP1 release bioactive TGF-β *in vitro*. Finally, we also identified the ability of GzmB to promote a direct TGF-β activation through the proteolysis of fibroblast-secreted proteins, independent of LAP degradation. Altogether, through the cleavage of specific ECM molecules including decorin and LTBP1, mast-cell derived GzmB may contribute to latent TGF-β activation and to the fibrotic response observed in KS and HS.

## Results

### GzmB accumulates in KS and HS

**Figure 1:**
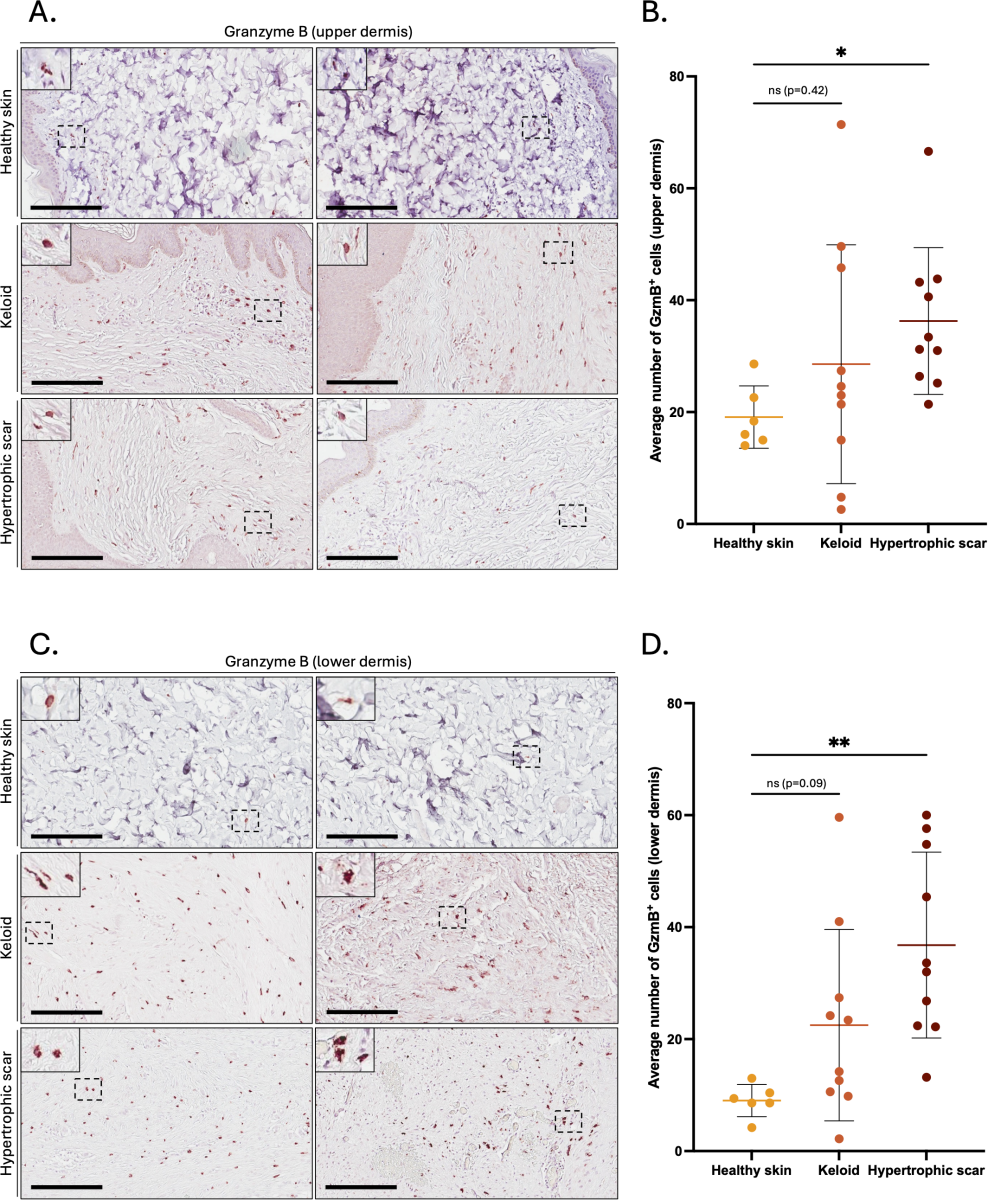
Granzyme B positive cells in the dermis of keloids and hypertrophic scars. **(A, C)** GzmB immunostaining performed on the upper **(A)** and lower **(C)** skin dermis of healthy controls and patients with keloids or hypertrophic scars. The inset shows high magnification of regions of interest. Bars, 200 μm. **(B, D)** Quantification of the average number of GzmB^+^ cells in the upper **(B)** and lower **(D)** dermis of healthy skin controls (n=6, orange), keloids (n=10, pink) and hypertrophic scars (n=10, brown) samples. Results are represented as mean ± SD. *p < 0.05, **p < 0.005. ns, not significant.

GzmB was assessed in the skin of 10 KS (2M/8F, mean age 32), 10 HS (6M/4F, mean age 48), and 6 healthy controls (1M/5F, mean age 45) by immunohistochemistry (patient information available in **Table 1**). Compared to healthy skin, the number of GzmB-positive (GzmB^+^) cells were significantly higher in the upper (**Figures 1A, B**) and lower (**Figures 1C, D**) dermis of HS. While the number of GzmB^+^ cells in KS appeared non-significant compared to healthy skin controls, a trend in the accumulation of GzmB^+^ cells was identified in the lower dermis (p=0.09, **Figure 1D**). No significant differences in the number of GzmB^+^ cells were observed between KS and HS.

**Table 1:** Individual patient information.

Pathology	Sex	Age	Site	GzmB^+^ cells upper/lower dermis	MCT^+^ cells upper/lower dermis	CD8^+^ cells upper/lower dermis	Decorin^+^ area (%age)	LTBP1 (DEJ)
Hypertrophic scar (HS, n=10)	M	28	Neck	31.2/32	46/33.8	16.6/7.6	2.44891624425	Absent
	M	35	Chest	66.6/57.6	86.6/74	17.8/7	4.82851807075	Absent
	F	39	Back	43.2/26.8	50.2/25.6	19.4/11.2	1.33604686225	Absent
	F	44	Breast	40.6/45.4	49.4/44.4	12/4.8	21.22335291	Present
	M	71	Brow	43.8/33.6	54.6/31.2	19.6/6	6.03769463125	Absent
	F	39	Shoulder	33.4/60	46.2/56.8	6.6/3.2	11.93677743275	Absent
	M	66	Back	31/13.2	21.8/13.8	1.4/2.4	0.041141697	Absent
	F	50	Abdomen	25.2/54.8	43/58.6	5.4/2	9.63497442825	Present
	M	59	Back	21.4/22.2	36/24.2	3.6/0.8	8.489850994	Absent
	M	52	Abdomen	26.4/22.4	49.4/36.8	3.8/1.8	2.1265761455	Absent
Keloid (KS, n=10)	M	27	Ear	23/12.6	25.2/14.6	5.4/7.4	0.286555085	Absent
	F	40	Shoulder	24.6/27.4	39.2/53.6	3.8/1.6	3.686345468	Absent
	F	22	Ear	2.6/10.6	13.6/13.6	14/4.2	0.11588579325	Present
	F	45	Shoulder	27.4/23.4	24.6/27.2	7/7	3.64769112125	Present
	F	28	Ear	15/9.8	29.2/12.2	7/0.4	0.258977943	Absent
	F	27	Ear	21.4/14.2	22.2/28.8	5.4/0.6	0.995697039	Present
	F	24	N/A	45.8/41	69.8/36	9.8/0.6	0.133266345	Absent
	M	61	Ear	71.4/59.6	83/46.6	11/3.4	0.81002639975	Absent
	F	25	Ear	49.6/24.2	77.4/35	5.4/0.6	10.56793153825	Absent
	F	21	Ear	4.8/2.2	11.6/7.6	2.8/2	5.42667168475	Present
Healthy skin (HC, n=6)	F	38	N/A	15/9.4	37.5/15.8	6.8/2.8	45.2025506305	Present
	F	40	N/A	16/8.6	36.6/23.8	6/2.6	40.6897406235	Present
	F	54	N/A	14/10.4	31/12	16.4/2.4	21.342699365	Present
	M	40	Trunk	22.6/13	33.2/11.2	30.2/7	35.3735474495	Present
	F	33	Trunk	18.4/4.2	34.4/18	13.8/5	26.469313207	Present
	F	67	Face	28.6/8.6	47.6/13.8	8.6/1.6	N/A	N/A

N/A, Not available.

### Mast cells are the primary source of GzmB in KS and HS

Based on the localization of GzmB in dermal areas characterized by immune cell infiltration, we next investigated the cell source of the protease by staining immune cell markers. Few CD8^+^ T-cells were detected in the upper and lower dermis of KS and HS (**Supplementary Figure 1A**), with no statistical differences compared to healthy skin controls (**Supplementary Figures 1B, C**). While a weak correlation was observed between the number of CD8^+^ and GzmB^+^ cells in the upper dermis of KS and HS (r=0.45, p=0.045, **Supplementary Figure 1D**), no correlation was observed in the lower dermis (r=0.17, p=0.49, **Supplementary Figure 1E**). This result indicates that CD8^+^ T-cells may not contribute to GzmB production in KS and HS.

**Figure 2:**
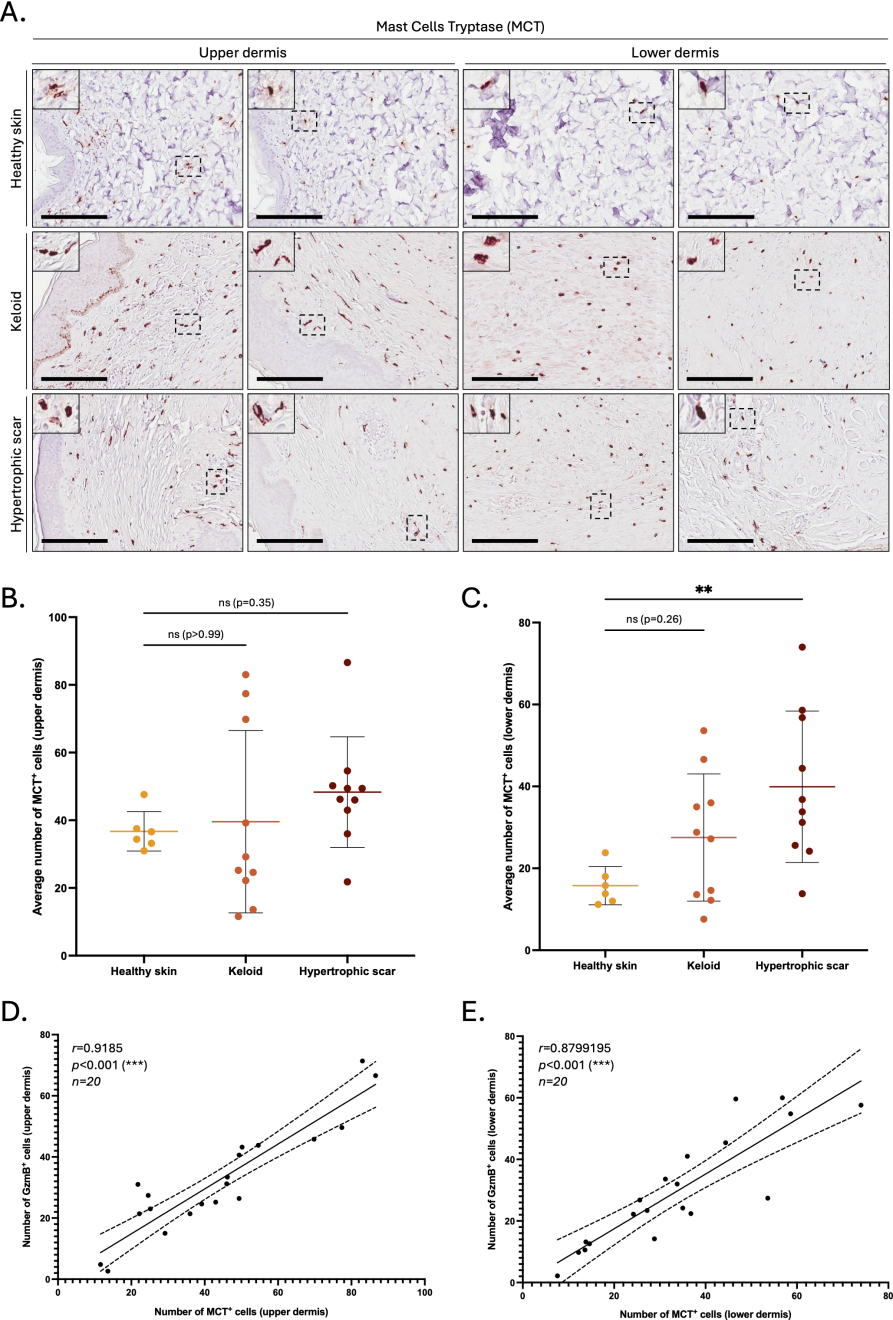
The number of MCT^+^ cells is positively correlated to the number of GzmB^+^ cells in the dermis of keloids and hypertrophic scars. **(A)** Mast Cell Tryptase (MCT) immunostaining performed on the upper and lower skin dermis of healthy controls and patients with keloids or hypertrophic scars. The inset shows high magnification of regions of interest. Bars, 200 μm. **(B, C)** Quantification of the average number of MCT^+^ cells in the upper **(B)** and lower **(C)** dermis of healthy skin controls (n=6, orange), keloids (n=10, pink) or hypertrophic scars (n=10, brown) samples. Results are represented as mean ± SD. **(D, E)** Correlation between the average number of GzmB^+^ and MCT^+^ cells in the upper **(D)** and lower **(E)** dermis of keloids or hypertrophic scars samples (n=20). **p < 0.01, ***p < 0.001. ns, not significant.

**Figure 3:**
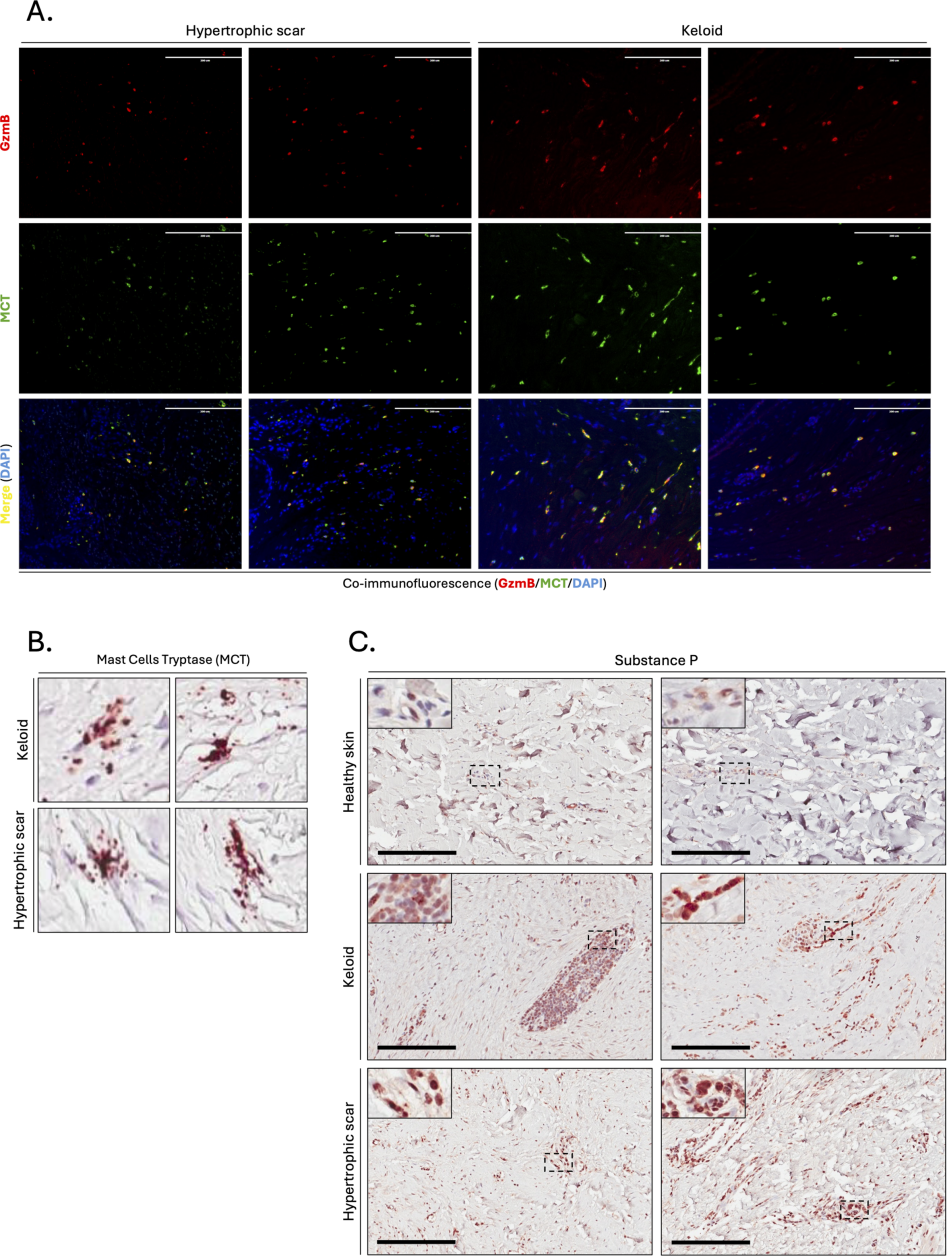
Mast cells are the primary source of GzmB in keloids and hypertrophic scars. **(A)** Co-immunofluorescence staining of GzmB (red) and MCT (green) in the skin dermis of keloids and hypertrophic scars. Merge shows overlap of the two channels and co-stained cells appear in yellow. Cell nuclei are stained with DAPI (blue). Scale, 200 μm. **(B)** MCT immunostaining demonstrating the presence of degranulating mast cells in the dermis of keloids and hypertrophic scars. **(C)** Substance P immunostaining performed on the skin dermis of healthy controls and patients with keloids or hypertrophic Scars. The inset shows high magnification of regions of interest. Bars, 200 μm.

In contrast, mast cells (defined by expression of mast cell tryptase, or MCT) significantly accumulated in the lower dermis of HS compared to healthy skin controls (**Figures 2A–C**). The numbers of MCT^+^ and GzmB^+^ cells in the upper (r=0.92, p>0.001, **Figure 2D**) and lower (r=0.88, p>0.001, **Figure 2E**) dermis of KS and HS (n=20) were significantly positively correlated, indicating mast cells as a major source of GzmB in these conditions. Co-immunofluorescence (**Figure 3A**) and tyramide signal amplification (TSA) (**Supplementary Figure 2**) assays revealed strong co-localization between MCT and GzmB, confirming mast cells as the main producer of the protease in KS and HS.

Since some of the mast cells in KS and HS exhibit active granule releas (**Figure 3B**), we immunostained skin dermis for Substance P, a neuropeptide previously identified as mediator of human mast cell degranulation (58). Increased presence of Substance P^+^ cells was observed in the dermis of KS and HS compared to healthy skin controls (**Figure 3C**), suggesting that mast cells release GzmB into the ECM of KS and HS through Substance P-mediated degranulation.

### Decorin and LTBP1 are reduced in KS and HS

**Figure 4:**
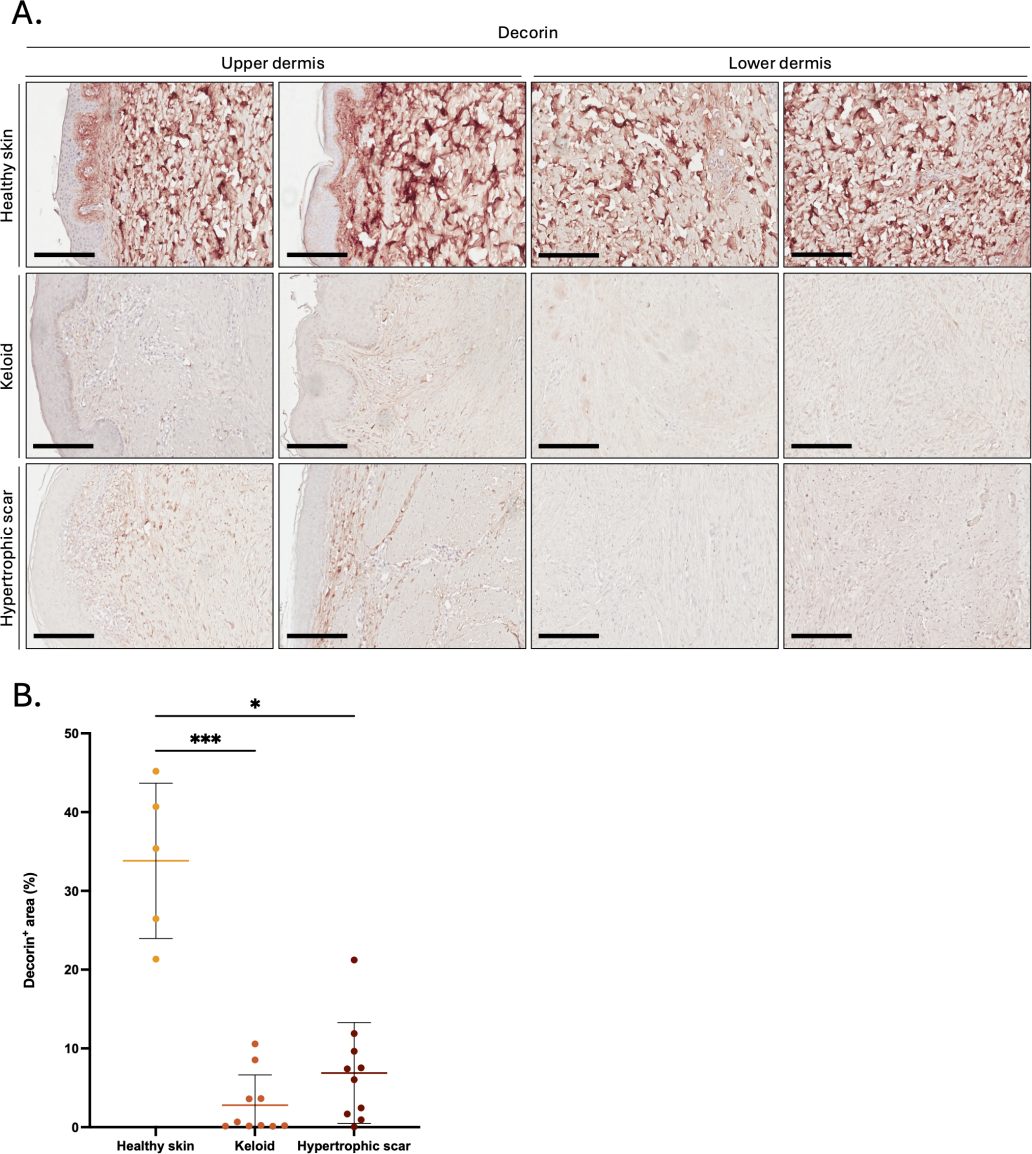
Decorin is reduced in the dermis of keloids and hypertrophic scars. **(A)** Decorin immunostaining performed on the upper and lower skin dermis of healthy controls and patients with keloids or hypertrophic scars. Bars, 200 μm. **(B)** Quantification of the decorin positive area (in %age of the total area) in the dermis of healthy skin controls (n=5, orange), keloids (n=10, pink) or hypertrophic scars (n=10, brown) samples. Results are represented as mean ± SD. *p < 0.05, ***p < 0.001.

Next, we investigated possible targets of mast-cell derived GzmB in KS and HS and first focused on decorin, an established extracellular GzmB substrate (57). In contrast to the abundance of decorin in healthy skin controls (**Figure 4A**), the intensity of decorin staining was significantly reduced in KS and HS (**Figure 4B**). While still partially detectable in the papillary dermis, decorin was almost completely absent from the reticular dermis of KS and HS (**Figures 4A, B**).

**Figure 5:**
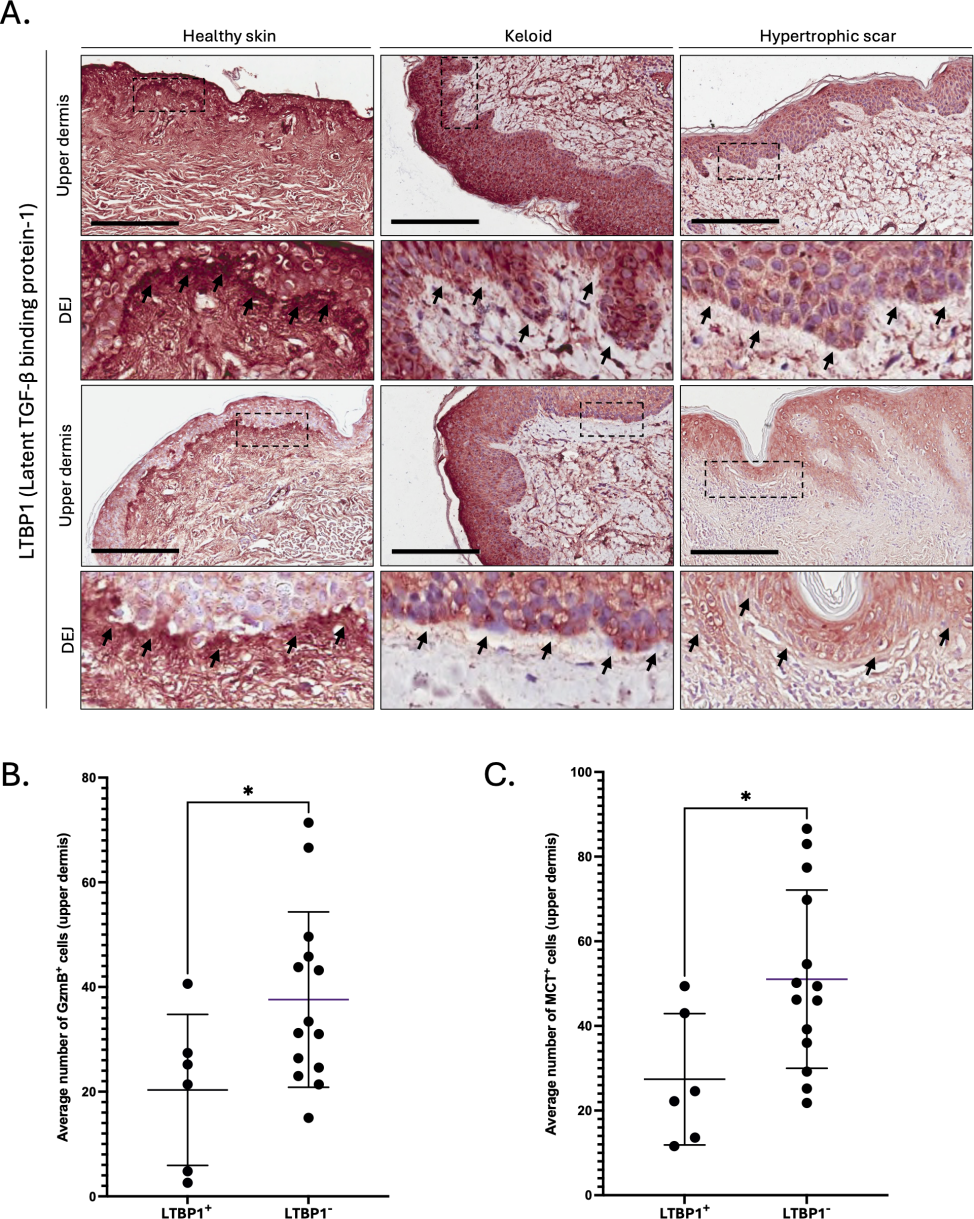
LTBP1 is reduced at the dermal epidermal junction of GzmB^+^ and MCT^+^ keloids and hypertrophic scars. **(A)** Latent TGF-β Binding Protein-1 (LTBP1) immunostaining performed on the upper dermis of healthy controls (n=5) and patients with keloids (n=10) or hypertrophic scars (n=10). The inset shows high magnification of the dermal epidermal junction (DEJ). Scale, 200 μm. **(B, C)** Average number of GzmB^+^ **(B)** and MCT^+^ **(C)** cells in the upper dermis of LTBP1^+^ (n=6) and LTBP1^-^ (n=14) keloids and hypertrophic scars. Results are represented as mean ± SD. *p < 0.05.

As decorin content was particularly low around the dermal epidermal junction (DEJ) of KS and HS, we investigated the levels of LTBP1, a large ECM protein located at the DEJ (59) and involved in the extracellular anchoring of latent TGF-β (9). Compared to healthy skin controls, reduced LTBP1 was observed at the DEJ of KS and HS (**Figure 5A**), with a more pronounced reduction in HS (absent from 8 out of 10 stained samples, **Supplementary Figure 3** and **Table 1**) than in KS (absent from 6 out of 10 stained samples, **Supplementary Figure 4** and **Table 1**). When pathological skin sections were divided into two groups based on the presence of LTBP1 at the DEJ (LTBP1^+^ versus LTBP1^-^), the average number of GzmB^+^ (**Figure 5B**) and MCT^+^ (**Figure 5C**) cells was significantly higher in the upper dermis of LTBP1^-^ skin lesions. Consequently, we hypothesized that GzmB may cleave LTBP1, resulting in its reduction at the DEJ of KS and HS.

### GzmB cleaves LTBP1 *in vitro*

**Figure 6:**
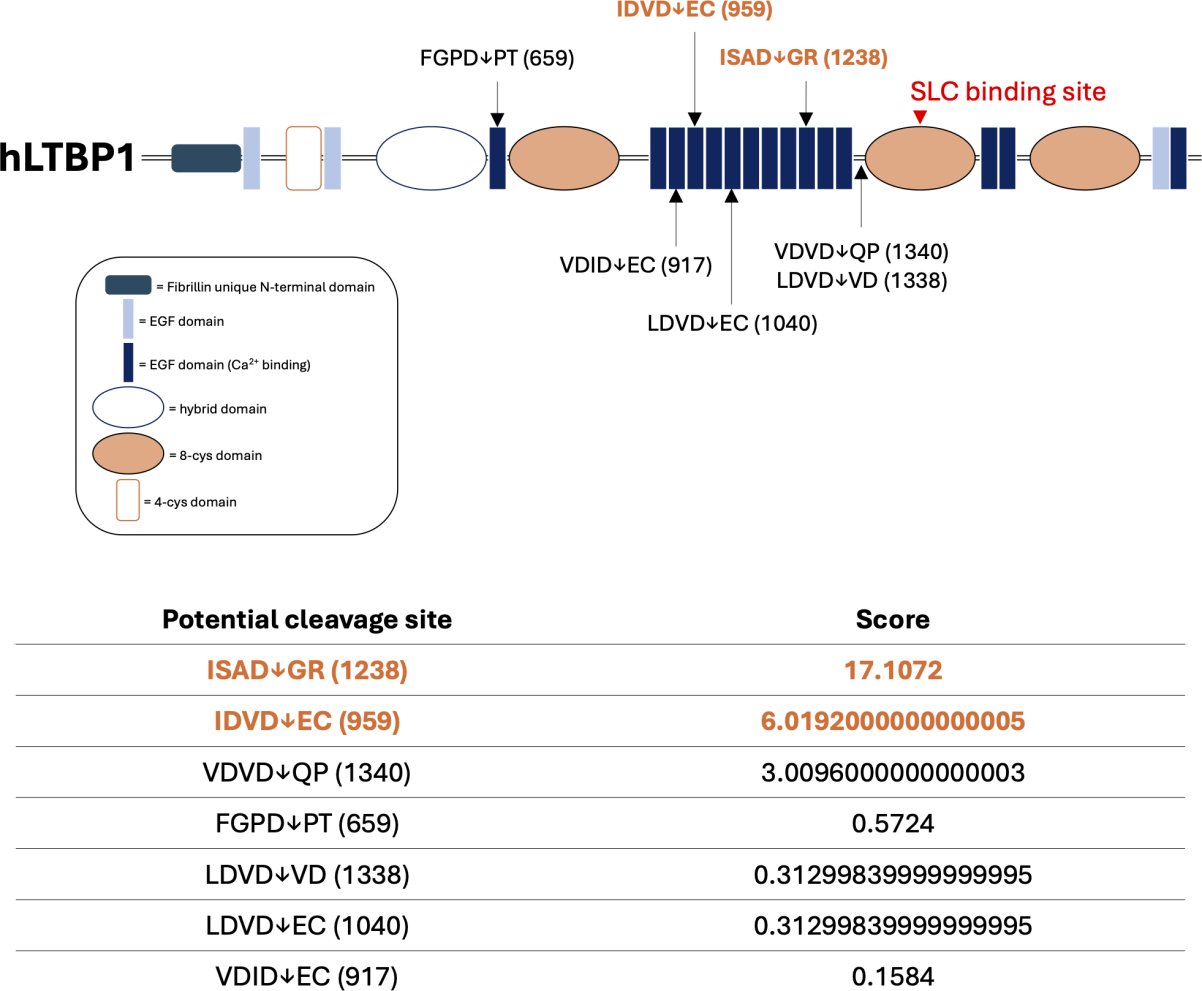
*In silico* prediction identifies LTBP1 as a potential new GzmB substrate. Schematic representation of predicted GzmB cleavage sites on human Latent TGF-β Binding Protein-1 (hLTBP1) amino-acid sequence. Letters represent the single amino acid code of the tetrapeptide preceding as well as the dipeptide following the expected cleavage site. Arrows represent the location of the expected cleavage site (after aspartic acid, D) and correspond to the amino acid location referenced between brackets. The score obtained using the GrabCas software is a readout of the cleavage susceptibility. The red arrowhead corresponds to the region responsible for the interaction between LTBP1 and the small latent complex (SLC). EGF, epidermal growth factor. Cys, cysteine.

Using the GrabCas software, a bio-informatic tool used to predict the cleavage of protease with aspase-like activities (60), we identified 7 potential GzmB cleavage sites in the LTBP1 amino-acid sequence (**Figure 6**). Those cleavage sites, mostly located on the central Ca^2+^ binding Epidermal Growth Factor (EGF)-domains of LTBP1, are compatible with the release of a C-terminal fragment containing the SLC binding site (**Figure 6**, red arrowhead).

**Figure 7:**
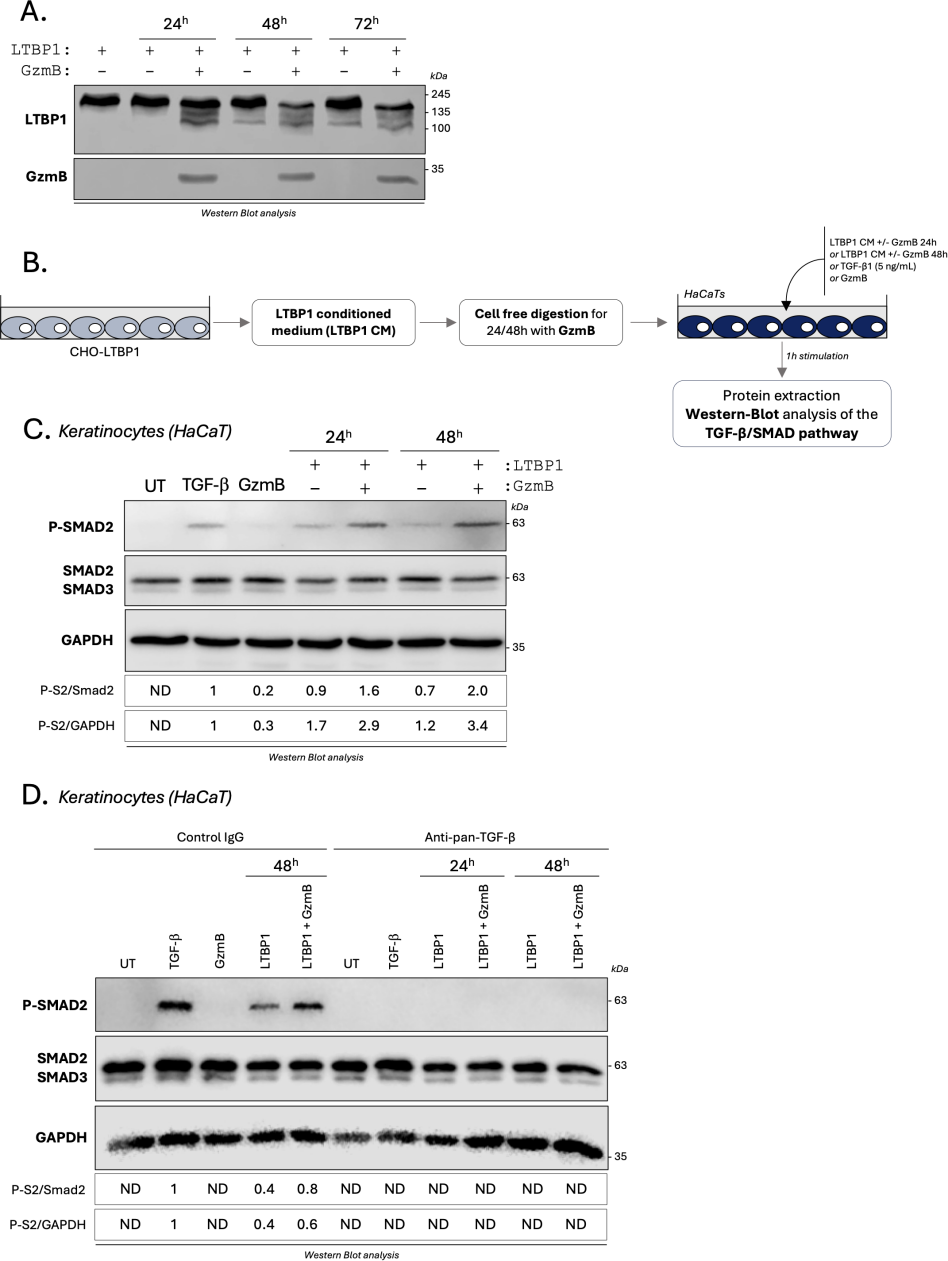
GzmB cleavage of LTBP1 *in vitro* activates TGF-β/Smad signaling pathway in epithelial cells. **(A)** LTBP1 conditioned media (from the same harvest) was precipitated, resuspended in GzmB digestion buffer, and incubated with (or without) 200 nM rhGzmB for several time point at 37°C and analyzed by western blot probing against LTBP1 (upper panel) and GzmB (lower panel). **(B)** Schematic representation of the experimental procedure used to investigate how GzmB digestion of LTBP1 modulates the TGF-β/Smad signaling pathway in keratinocytes (HaCaTs). **(C)** Western blot analysis showing P-Smad2, total Smad2/3 and GAPDH levels in HaCaTs cultured for 1h as described in **(B)**. **(D)** Western blot analysis showing P-Smad2, total Smad2/3 and GAPDH levels in HaCaTs cultured for 1h as described in **(B)** in presence of an anti-pan-TGF-β inhibitor or an isotype-matched control IgG. UT, untreated; ND, non-detectable.

Next, we investigated GzmB cleavage of LTBP1 in a cell free cleavage assay. Using CHO cells stably transfected with a plasmid encoding LTBP1 (CHO-LTBP1 (61)), we generated LTBP1-enriched conditioned media (LTBP1 CM). After protein precipitation, LTBP1 CM was incubated with GzmB for different time points followed by western blot analysis of the digestion products. Compared to non-digested controls, we identified a reduced LTBP1 molecular weight in the presence of GzmB (**Figure 7A**), indicating that GzmB can cleave LTBP1 from conditioned medium *in vitro*. A morphological switch towards fibroblastic-like features was also observed in CHO-LTBP1 cultured in presence of 100 nM GzmB (**Supplementary Figure 5A**, arrowheads). This partial epithelial to mesenchymal transition (EMT) phenotype was not observed in CHO-LTBP1 treated with PBS or with a lower GzmB concentration (50 nM). Consequently, we hypothesized that GzmB may modulate latent TGF-β bioavailability through the cleavage of LTBP1, thereby regulating the induction of EMT in epithelial cells (**Supplementary Figure 5A**).

### GzmB cleavage of LTBP1 activates TGF-β/Smad signaling pathway in epithelial cells

To characterize the functional consequences of GzmB-dependent LTBP1 cleavage on latent TGF-β activation, LTBP1 CM was digested with GzmB and subsequently used to treat human keratinocytes for 1 h (**Figure 7B**, optimized in **Supplementary Figure 5B**). Treatments with active TGF-β1 or GzmB alone were used as positive and negative controls respectively, and Smad2 phosphorylation was assessed in keratinocyte lysates as a readout for activated TGF-β1 signaling (**Figure 7B**). To ensure that the observed effect was dependent on LTBP1 cleavage and independent of decorin degradation, the absence of decorin from LTBP1 CM was confirmed by western blot analysis (**Supplementary Figure 5C**).

In contrast to the faint Smad2 phosphorylation observed in untreated or GzmB-treated keratinocytes (**Figure 7C**), treatment of HaCaT keratinocytes with GzmB-digested LTBP1 CM resulted in an increased Smad2 phosphorylation compared to the non-digested LTBP1 CM, with a 1.7-fold increase for 24h and 2.8-fold for 48h digestion (**Figure 7C**). To confirm the involvement of TGF-β in this process, we performed the same experiments in the presence of a pan-TGF-β inhibitor or an isotype match control antibody (**Figure 7D**). While Smad2 phosphorylation was unaffected by the control antibody, treatment with the pan-TGF-β inhibitor completely abolished Smad2 phosphorylation in all conditions (**Figure 7D**). This observation confirmed that GzmB can promote TGF-β dependent Smad2 phosphorylation through the generation of LTBP1 fragments. At the used concentration (200 nM), GzmB alone does not promote Smad2 phosphorylation in keratinocytes (**Figures 7C, D**).

To mitigate the effect of growth factors in the culture medium, we produced and purified recombinant human (rh)LTBP1 from conditioned medium of stably transfected CHO cells (**Supplementary Figure 6A**). Using cell free cleavage assay, we confirmed the ability of GzmB to cleave purified rhLTBP1 *in vitro* (**Supplementary Figure 6A**). Keratinocytes treated with rhLTBP1 for 1h demonstrated an increased and dose dependent Smad2-phosphorylation compared to non-treated controls (**Supplementary Figure 6B**). Pre-digestion of rhLTBP1 by GzmB resulted in a 2-fold increased Smad2 phosphorylation compared to the non-digested rhLTBP1 condition in keratinocytes (**Supplementary Figure 6C**), thus confirming the results obtained using CHO-derived LTBP1 CM (**Figure 7**).

As an additional indicator for TGF-β1 activity, p38 and p44/42 non-canonical TGF-β signaling pathways were also assessed in epithelial cells (62). Compared to the non-treated condition, LTBP1 CM promoted the phosphorylation of MAPK p38 (**Supplementary Figure 7A**) and p44/42 (**Supplementary Figure 7B**). Despite the ability of GzmB to directly activate both MAPK pathways, we observed no differences in p38 phosphorylation (**Supplementary Figure 7A**) as well as a reduction of p44/42 phosphorylation (**Supplementary Figure 7B**) for LTBP1 CM pre-digested with GzmB. Additionally, the LTBP1/GzmB-mediated MAPK activation was still detectable in the presence of the pan-TGF-β inhibitor (**Supplementary Figures 7C, D**), suggesting that this effect may be independent of TGF-β and directly mediated by LTBP1.

### GzmB cleavage of LTBP1 activates TGF-β/Smad signaling pathway in dermal fibroblasts

**Figure 8:**
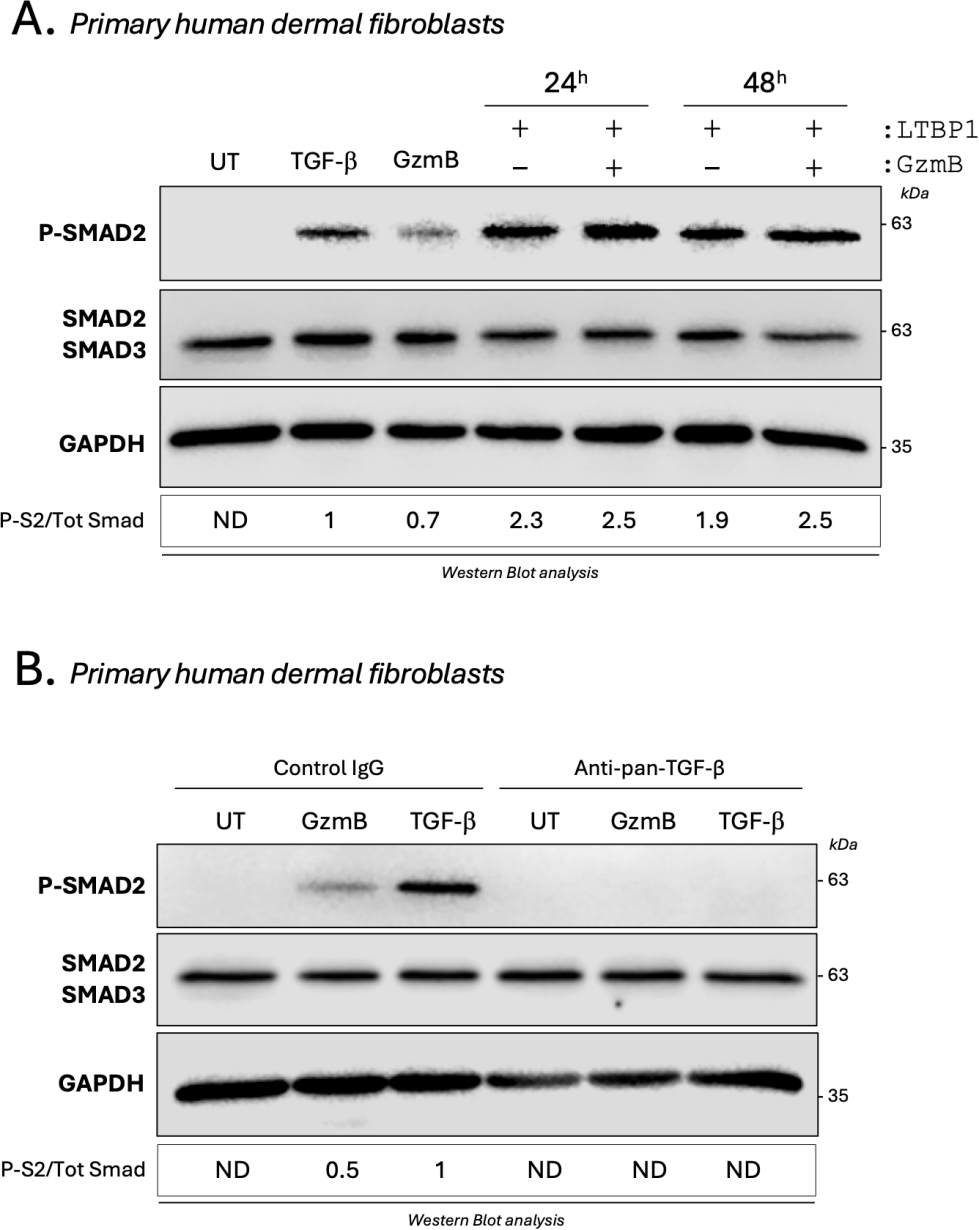
GzmB promotes a direct and TGF-β dependent Smad2 phosphorylation in dermal fibroblasts. **(A)** Western blot analysis showing P-Smad2, total Smad2/3 and GAPDH levels in primary human dermal fibroblasts cultured for 1h as described in **Figure 7B**. **(B)** Western blot analysis showing P-Smad2, total Smad2/3 and GAPDH levels in untreated (UT) primary human dermal fibroblasts or primary human dermal fibroblasts treated for 1h with TGF-β1 (5 ng/mL) or 200 nM GzmB in presence of an anti-pan-TGF-β inhibitor or an isotype-matched control IgG. UT, untreated; ND, non-detectable.

Based on the fibroproliferative nature of KS and HS and because fibroblasts are the primary source of ECM molecules in fibrosis, the same experiments were performed using primary human dermal fibroblasts. While only a small increase in Smad2 phosphorylation was observed in primary dermal fibroblasts cultured with GzmB-digested LTBP1 CM compared with non-digested LTBP1 CM (**Figure 8A**), a 2-fold increase in Smad2 phosphorylation was observed when primary dermal fibroblasts were treated with GzmB-digested rhLTBP1 compared to the non-digested protein (**Supplementary Figure 8**). Together, these findings show that GzmB-dependent LTBP1 cleavage activates TGF-β signaling in dermal fibroblasts.

### GzmB promotes TGF-β activation through the cleavage of one or more fibroblast secreted protein(s)

Unexpectedly, and in opposition to what was previously observed in keratinocytes (**Figures 7C, D**), a marked Smad2 phosphorylation was detectable in primary dermal fibroblasts treated with 200 nM GzmB alone (**Figure 8A**). This phosphorylation was completely abolished in the presence of the pan-TGF-β inhibitor (**Figure 8B**), indicating that GzmB itself can directly activate latent TGF-β secreted by dermal fibroblasts.

**Figure 9:**
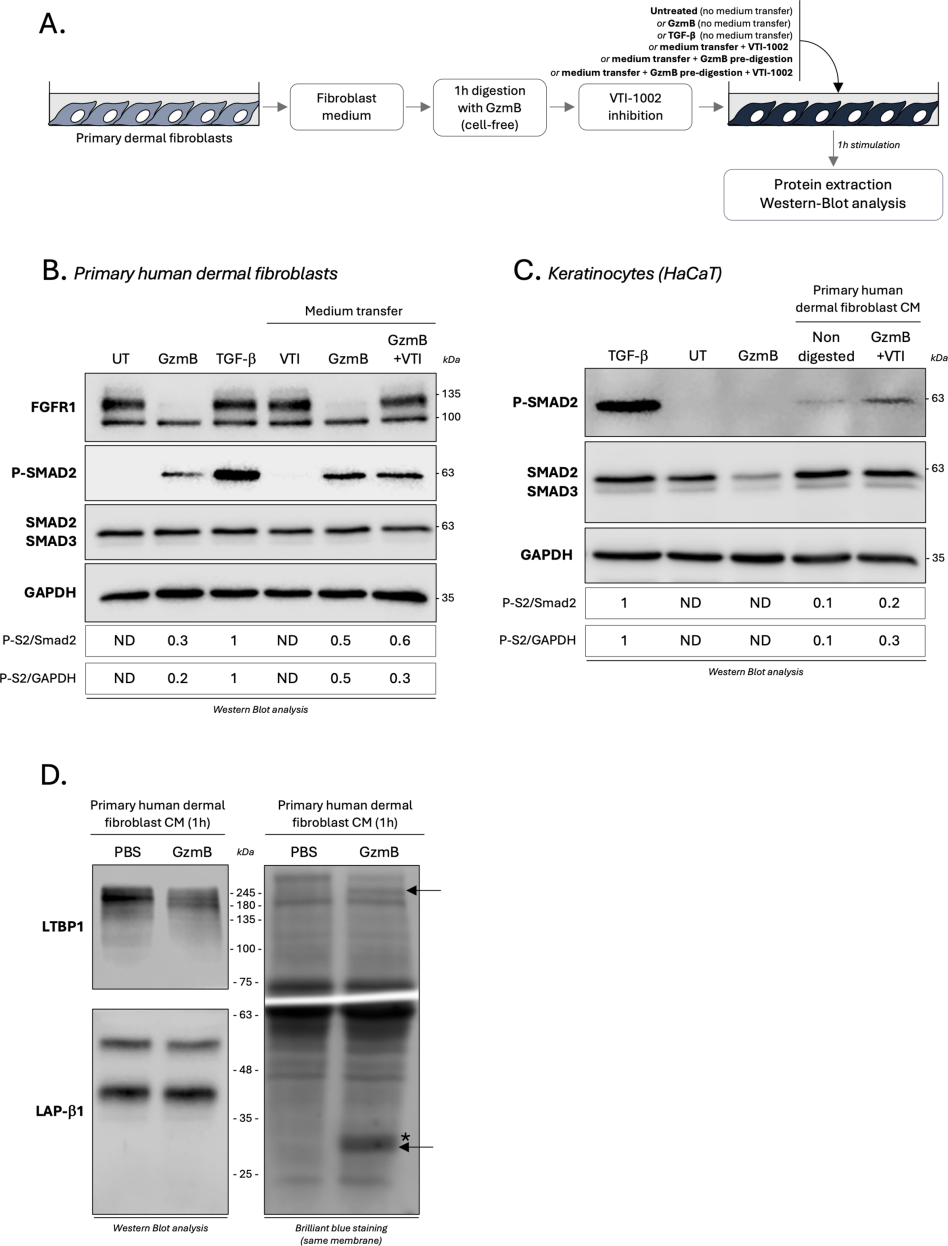
GzmB promotes TGF-β activation through the cleavage of one or more fibroblast secreted protein(s). **(A)** Schematic representation of the experimental procedure used to investigate how GzmB proteolysis of primary dermal fibroblast CM modulates the TGF-β/Smad signaling pathway. **(B)** Western blot analysis showing FGFR1, P-Smad2, total Smad2/3 and GAPDH levels in primary dermal human fibroblasts cultured for 1h as described in **(A)**. **(C)** Western blot analysis showing P-Smad2, total Smad2/3 and GAPDH levels in HaCaTs cultured for 1h as described in **(A)**. **(D)** Western blot analysis demonstrating the ability of GzmB to cleave LTBP1 (top left panel) but not LAP-β1 (bottom left panel) from primary dermal fibroblast CM after 1h incubation. The right panel represent brilliant blue staining of the same membrane. *De novo* generated fragments are indicated by a black arrow and GzmB in indicated by a (*). UT, untreated; ND, non-detectable; CM, conditioned medium.

We next investigated whether this direct activation of the TGF-β/Smad signaling pathway was regulated by GzmB-dependent proteolysis of secreted proteins. To do so, primary human dermal fibroblasts were starved for 24h to generated fibroblast-derived CM enriched in secreted proteins. In a cell free system, fibroblast CM was incubated for 1h with GzmB to generate protein fragments. Then, and in order to mitigate the direct effect of GzmB, the protease was inhibited using its specific extracellular inhibitor VTI-1002 (**Figure 9A**). The digested fibroblast medium, thus containing protein fragments and inactivated GzmB, was used to treat dermal fibroblasts or keratinocytes for 1h followed by western blot analysis of the TGF-β/Smad signaling pathway (**Figure 9A**). A no inhibitor control, as well as a VTI-1002 alone control, were also included.

While VTI-1002 inhibited GzmB proteolytic activity as demonstrated by the nullification of fibroblast growth factor receptor 1 (FGFR1) cleavage, a strong Smad2 phosphorylation in primary dermal fibroblast was still detectable after GzmB inhibition (**Figure 9B**). Similar results were obtained using keratinocytes, with a 2- to 3-fold increased Smad2 phosphorylation when cultured in the presence of primary dermal fibroblast CM pre-digested with GzmB compared to non-digested controls (**Figure 9C**). As previously reported (**Figures 7C, D**), GzmB alone does not promote Smad2 phosphorylation in keratinocytes (**Figure 9C**).

Using dermal fibroblast CM digested for 1h with GzmB (cell free system), we confirmed the ability of GzmB to cleave fibroblast-derived LTBP1 by immunoblotting (**Figure 9D**, left panel). We also identified the presence of two newly generated degradation products in the CM of fibroblasts incubated with GzmB (**Figure 9D**, right panel, black arrows). As the intensity and molecular weight of the LAP-β1 pro-peptide (a component of the SLC) remained the same after 1h incubation with GzmB (**Figure 9D**, left panel), these results suggest a mechanism whereby GzmB promotes indirect latent TGF-β activation from fibroblast medium through the cleavage of one (or more) unidentified secreted protein(s).

## Discussion

Tethered to the ECM as part of the SLC or the LLC, TGF-β is activated extracellularly in a tightly regulated process that involves ECM molecules, cell surface integrins, and/or extracellular proteases. While increased TGF-β signaling in fibrosis is well-established (28, 29), little is known about the molecular mechanisms involved in the regulation of its extracellular activation in KS and HS. Results from this study suggest that extracellular GzmB contributes to KS and HS pathology through the cleavage of ECM molecules involved in the regulation of latent TGF-β activation.

In the dermis of patients with HS, a significant accumulation of GzmB-expressing cells, especially mast cells, was observed. Compared to healthy skin controls, increased Substance P, a mediator of human mast cell degranulation, was also evident in both KS and HS (58). While the cell source of Substance P remains to be elucidated, these findings suggest that GzmB^+^ mast cells are a key source of extracellular GzmB in KS and HS. Since mast cells do not express perforin (63), it is possible that Substance P-mediated mast cell degranulation results in the accumulation of GzmB in the extracellular milieu. Consequently, as previously reported in other dermatological conditions (49–51, 54), GzmB may contribute to KS and HS through perforin-independent extracellular mechanisms. Nevertheless, extracellular release of GzmB after Substance P-mediated mast cell degranulation will have to be confirmed experimentally.

In addition to confirming the reduction of decorin in the dermis of KS (37, 38) and HS (39, 40), we reported the reduction of LTBP1 at the DEJ of KS and HS and confirmed its cleavage by GzmB *in vitro*. While we cannot exclude that LTBP1 reduction observed in KS and HS is due to its downregulation at a transcriptional level or to its degradation by other proteases (notably Bone Morphogenic Protein (BMP) 1 (64) and membrane-type 1 MMP (MT1-MMP) (23)), the abundance of GzmB^+^ mast cells in the upper dermis of LTBP1-negative lesions implicates GzmB as a potential contributor of its degradation in human samples.

*In vitro*, GzmB-dependent cleavage of LTBP1 promoted Smad2 phosphorylation in epithelial cells, most likely by generating LTBP1 fragments with increased ability to activate TGF-β. This mechanism, similar to the one previously identified for BMP-1 cleavage of thrombospondin-1 (65), might explain the partial EMT observed in CHO-LTBP1 cells treated with GzmB. A deeper analysis of the induced TGF-β/Smad signaling pathway, notably the downstream transcription of TGF-β targeted genes involved in EMT or ECM molecule synthesis, will help to decipher the precise roles of the GzmB/LTBP1 axis in latent TGF-β activation. It is also worth noting that fibrillin-1 and fibronectin, two LTBP1-interacting glycoproteins involved in the extracellular tethering of the LLC (9), are established GzmB substrates validated *in vivo* in different disease models (52, 66). Consequently, and in addition to the direct cleavage of LTBP1, GzmB may impair the extracellular anchoring of LTBP1 to the fibrillin-/fibronec,n-rich matrix, with potential consequences in biomechanical properties of KS and HS lesions. A similar mechanism has been notably reported in burn wounds, where GzmB dependent cleavage of decorin impaired wound remodeling through the modification of collagen bundle organization (54). This hypothesis can be evaluated by performing atomic force microscopy in KS and HS samples (67).

In the present study, epithelial cells and fibroblasts responded differently to GzmB stimulation. While no Smad2 phosphorylation was observed in keratinocytes treated with the protease, TGF-β-dependent Smad2 phosphorylation was observed in primary dermal fibroblasts treated with GzmB. In addition to confirming the cleavage of fibroblast-derived LTBP1, we also identified the presence of two protein fragments in the conditioned media of fibroblast digested with GzmB. Consequently, and keeping in mind that GzmB does not cleave the LAP pro-peptide, it is plausible that GzmB contributes to latent TGF-β activation through the cleavage of fibroblast-secreted ECM molecules that are not produced by keratinocytes. Such mechanism could involve several ECM molecules involved in TGF-β activation and already identified as GzmB substrates, such as LTBP1, decorin (57), thrombospondin-1 (68), fibrillin-1 (66), and/or tenascin-C (69). While this hypothesis will require *in vitro* and *in vivo* experimental validation, GzmB-mediated TGF-β activation also needs to be re-evaluated in primary cells extracted from KS and HS, as fibroblasts from these two conditions exhibit increased sensitivity to TGF-β stimulation compared to healthy dermal fibroblasts (33–36). Using patient-derived primary cells will also help us to decipher whether LTBP1 expression is altered at the transcriptional level in KS and HS.

Over the past 10 years, TGF-β-based monotherapies have only demonstrated mild successes in the context of KS and HS (5). This may be due to the fact that while TGF-β1 and β2 isoforms are pro-fibrotic, TGF-β3 is anti-fibrotic (70, 71). As GzmB is, to our knowledge, exclusively involved in TGF-β1 activation, targeting the protease in the context of wound healing might be a promising strategy to specifically tackle TGF-β pro-fibrotic effects. Of note, topical inhibition of GzmB reduced scarring and promoted the healing of burn wounds (54), and reduced ECM accumulation as well as myofibroblast differentiation in a model of cardiac fibrosis (72). While the burn model is relevant in the context of studying HS, a role for GzmB in KS has never been assessed *in vivo*. Such an approach is challenged by the difficulty of implementing a physiologically relevant KS model in mice, which usually requires the implantation of human keloid explants (or cells derived from human KS samples) in immunocompromised animals (73). Nevertheless, performing this model in a GzmB deficient background will be the next step in understanding the precise roles of the protease in the development of KS.

Altogether, this work provides a novel mechanism for GzmB-induced TGF-β activation in the context of scarring and fibrosis.

## Materials and methods

### Study approvals

Use of human biospecimens for this study was approved by the University of British Columbia (Clinical research ethics board no: H23-01301). Skin biopsies from healthy skins as well as keloid and hypertrophic scars were collected and provided by Dr. Richard Crawford (Department of Dermatology, University of British Columbia, Vancouver, BC, Canada). Patient information is available in **Table 1**.

### Immunohistochemistry, tyramide signal amplification and co-immunofluorescence

Immunostaining was performed as previously established (47, 49). Briefly, after dewaxing and rehydration, antigen retrieval was performed in 10 mM sodium citrate buffer (Sigma) containing 0.05% Tween-20 (Sigma-Aldrich) pH 6 for 20 min in a steamer. Sections were then cooled down for 20 min at room temperature and endogen peroxidases were quenched by 3% H_2_O_2_ (v/v). Non-specific sites were then blocked for 30 min at RT followed by primary antibody incubation overnight at 4°C (diluted in blocking solution). The next day, after 30 min to 1h incubation with the secondary antibody (diluted in blocking solution), HRP was coupled with the biotinylated secondary antibody for 30 min using the VECTASTAIN Elite ABC-Peroxidase kit (Vector laboratories).

#### Immunohistochemistry

Revelation was performed using the Vector NovaRED Substrate Kit (Vector laboratories). Sections were counterstained with modified Harris Hematoxylin (Sigma-aldrich), blued in 0.5% Lithium Carbonate and mounted using the xylene-based Cytoseal™ 60 medium (Epredia). Slides were scanned using the APERIO CS2 slide scanner (Leica).

#### Tyramide signal amplification (TSA)

Revelation was performed according to the manufacturer recommendations. First revelation was performed using the Green Fluorescein Amplification Reagent (Akokabio) diluted at 1:50 in TBS-T for 5 min at RT. Avidin and Biotin free sites were then blocked for 15 min each at RT (Akokabio), followed by 30 min blocking and overnight incubation at 4°C with the second primary antibody (diluted in blocking solution). The next day, after secondary antibody incubation and HRP coupling, the second revelation was performed using the Red Fluorescein Amplification Reagent (Akokabio) diluted at 1:50 in TBS-T for 5 min at RT. Nuclei were then stained for 3 min with DAPI (1:1,000 in PBS) and sections were mounted using the water-based Hydromount medium (Electron Microscopy Sciences). Pictures were taken using a fluorescence inverted microscope (EVOS FL, Life Technology) and analyzed using the FIJI software (SciJava).

#### Co-Immunofluorescence

After de-waxing, rehydration and antigen retrieval, sections were permeabilized for 10 min with 0.5% tritton-X100 (FisherBiotech) and blocked in 5% FBS for 30 min prior co-incubation overnight at 4°C with both primary antibodies (diluted in blocking solution). The next day, after 1h of co-incubation with A593/A488-coupled secondary antibodies, nuclei were stained for 3 min with DAPI (1:1,000 in PBS) and sections were mounted using the water-based Hydromount medium (Electron Microscopy Sciences). Pictures were taken using a fluorescence inverted microscope (EVOS FL, Life Technology) and analyzed using the FIJI software (SciJava). Blocking conditions as well as primary and secondary antibodies dilution are detailed in **Table 2**.

**Table 2:** List of primary and secondary antibodies used for immunohistochemistry (IHC), tyramide signal amplification (TSA), co-immunofluorescence (Co-IF) and Western blotting (WB).

Application	Primary antibody	Blocking solution	Primary antibody reference	Primary antibody dilution and buffer	Secondary antibody	Secondary antibody dilution and buffer
IHC/TSA	Rabbit anti-human GzmB	10% goat serum in TBS	Abcam (Ab4059)	1/600 in TBS + 10% goat serum	Biotinylated goat anti-rabbit	1/350 in TBS, 10% goat serum
IHC/TSA	Mouse anti-human Mast Cell Tryptase	5% horse serum in TBS	Biorad (MCA1438)	1/10,000 in TBS + 5% horse serum	Biotinylated horse anti-mouse	1/350 in TBS, 5% horse serum
IHC	Rabbit anti-human LTBP1	10% goat serum in TBS	Proteintech (26855-1-AP)	1/50 in TBS + 10% goat serum	Biotinylated goat anti-rabbit	1/350 in TBS, 10% goat serum
IHC	Mouse anti-human CD8	5% horse serum in TBS	Dako (M7103)	1/100 in TBS + 5% horse serum	Biotinylated horse anti-mouse	1/350 in TBS, 5% horse serum
IHC	Goat anti-human Decorin	10% rabbit serum in TBS	R&D (AF-143)	1/1,000 in TBS + 10% rabbit serum	Biotinylated rabbit anti-goat	1/350 in TBS, 10% rabbit serum
IHC	Rabbit anti-human Substance P	10% goat serum in TBS	Invitrogen (PA5-106934)	1/100 in TBS + 10% goat serum	Biotinylated goat anti-rabbit	1/350 in TBS, 10% goat serum
Co-IF	Rabbit anti-human GzmB	5% FBS in PBS	Abcam (Ab4059)	1/100 in PBS + 5% FBS	A594-conjugated donkey anti-rabbit	1/350 in PBS, 5% BSA
Co-IF	Mouse anti-human Mast Cell Tryptase		Biorad (MCA1438)	1/1,000 in PBS + 5% FBS	A488-conjugated donkey anti-mouse	1/350 in PBS, 5% BSA
WB	Rabbit anti-human LTBP1	10% milk in TBS-0.1% Tween	Proteintech (26855-1-AP)	1/1,500 in TBS-0.1% Tween + 5% milk	HRP-conjugated goat anti-rabbit	1/10,000 in TBS-0.1% Tween, 10% milk
WB	Mouse anti-human GzmB		BD pharmingen (550558)	1/4,000 in TBS-0.1% Tween + 5% milk	HRP-conjugated goat anti-mouse	1/10,000 in TBS-0.1% Tween, 10% milk
WB	Rabbit anti-human Phospho-Smad2 (Ser465/467)		Cell signaling (mAb#3108)	1/1,000 in TBS-0.1% Tween + 5% milk	HRP-conjugated goat anti-rabbit	1/5,000 in TBS-0.1% Tween, 10% milk
WB	Rabbit anti-human total smad2/3		Cell signaling (mAb#3102)	1/500 in TBS-0.1% Tween + 5% milk	HRP-conjugated goat anti-rabbit	1/2,500 in TBS-0.1% Tween, 10% milk
WB	Rabbit anti-human GAPDH		Cell signaling (mAb#2118)	1/1,000 in TBS-0.1% Tween	HRP-conjugated goat anti-rabbit	1/10,000 in TBS-0.1% Tween, 5% milk
WB	Mouse anti-human Phospho-p38 (Thr180/Tyr182)		Cell signaling (mAb#9216)	1/2,000 in TBS-0.1% Tween + 5% milk	HRP-conjugated goat anti-mouse	1/10,000 in TBS-0.1% Tween, 10% milk
WB	Rabbit anti-human total p38		Cell signaling (mAb#9212)	1/1,000 in TBS-0.1% Tween + 5% milk	HRP-conjugated goat anti-rabbit	1/10,000 in TBS-0.1% Tween, 10% milk
WB	Mouse anti-human Phospho-p44/42		Cell signaling (mAb#9106)	1/2,000 in TBS-0.1% Tween + 5% milk	HRP-conjugated goat anti-mouse	1/10,000 in TBS-0.1% Tween, 10% milk
WB	Rabbit anti-human total p44/42		Cell signaling (mAb#9102)	1/1,000 in TBS-0.1% Tween + 5% milk	HRP-conjugated goat anti-rabbit	1/10,000 in TBS-0.1% Tween, 10% milk
WB	Rabbit anti-human FGF Receptor 1 (D8E4)		Cell signaling (mAB#9740)	1/1,000 in TBS-0.1% Tween + 5% milk	HRP-conjugated goat anti-rabbit	1/10,000 in TBS-0.1% Tween, 10% milk
WB	Goat an,-human LAP (TGF-β1)		R&D (AF-246-NA)	1/100 in TBS-0.1% Tween + 5% milk	HRP-conjugated donkey anti-goat	1/2,000 in TBS-0.1% Tween, 10% milk
WB	Goat anti-human Decorin		R&D (AF-143)	1/2,000 in TBS-0.1% Tween + 5% milk	HRP-conjugated donkey anti-goat	1/5,000 in TBS-0.1% Tween, 10% milk

### Prediction of GzmB cleavage sites in human LTBP1

The protein sequence of human LTBP1 was obtained from Uniprot (assession no.: Q14766-1) and assessed for potential GzmB cleavage sites using GraBCas (v1.0) software with a cutoff of 0.1 (60). Cleavage sites were mapped to their respective regions in the full-length LTBP1 according to the domain organization available on Uniprot and displayed in (9).

### Cell culture and generation of LTBP1 conditioned medium

Kera,nocytes (HaCaTs) and primary human dermal fibroblasts were maintained in complete Dulbecco modified Eagle’s medium (DMEM) containing 10% (v/v) Fetal Bovine Serum (FBS, Gibco) and 1% (v/v) Penicillin-Streptomycin (PS, Sigma). Chinese Hamster Ovary (CHO) cells stably transfected with the plasmid encoding LTBP1 (hereafter named CHO-LTBP1) was a gift from Boris Hinz (University of Toronto, Ontario, Canada) and were maintained in DMEM containing 10% (v/v) FBS, 1% (v/v) PS and selected with 1 mg/mL Geneticin (G418 sulfate, Agilent technology). To generate LTBP1-conditioned medium, CHO-LTBP1 were cultured to over-confluency for 3 weeks under serum deprivation and culture medium was collected 3 times a week. Conditioned media enriched in LTBP1 were cleared of cellular debris by centrifugation at 300 g for 10min at room temperature and stored at -20°C. Presence of LTBP1 in CM was assessed by immunoblotting.

### Recombinant human LTBP1 production and purification

CHO cells transfected with the pSecTag/FRT/V5-His-TOPO vector encoding LTBP1-EGFP (61) were donated from Boris Hinz (University of Toronto, Ontario, Canada) and were maintained in complete DMEM containing 10% (v/v) Fetal Bovine Serum (FBS, Gibco), 1% (v/v) Penicillin-Streptomycin (PS, Sigma) and 1 mg/mL Geneticin (G418, Gibco). Stably transfected CHO cells were culture to overconfluence for 2 weeks under serum deprivation. Culture medium was collected 3 times a week, centrifuged at 300g for 5 minutes to remove cellular debris and stored at -20°C. Prior purification, LTBP1-EGFP enriched conditioned medium was dialyzed 3 times (twice for 2 hours, then overnight) at 4°C in PBS using 6-8 kDa dialysis membrane (SpectrumLabs). LTBP1-EGFP conditioned medium was then directly applied on HisTrap HP His Tag protein purification columns (5 mL, Cytiva Life Sciences) using the ÄKRA Start Chromatography System (Cytiva Life Sciences). Column was washed using PBS containing 20 mM imidazole and eluted using 300 mM imidazole diluted in PBS. Fractions enriched in LTBP1-EGFP were analyzed by Brilliant Blue Staining (MiliporeSigma) and immunoblotting, pulled together, and dialyzed 3 times overnight at 4°C in PBS, PBS containing 0.2% (v/v) chloroform, and PBS before storage at -80°C. LTBP1-EGFP concentration was determined using the Pierce BCA protein Assay Kit (Thermo Fisher Scientific).

### *In vitro* cell free digestion assay

#### Conditioned medium

1 mL of LTBP1 CM from the same set of harvest was precipitated with 10% final Tri-chloroacetic acid (Sigma-aldrich) for 20 min on ice and proteins were pelleted by centrifugation at 14,324 g for 10 min at 4°C. After removal of the supernatant, protein pellets were washed twice with cold 100% ethanol (VWR) before being resuspended in 50 μL of GzmB digestion buffer (50 mM Tris in PBS, pH 7.4). 200 nM GzmB (Bon Opus) or an equivalent volume of PBS was then added to the samples and incubated at 37°C for 24, 48 or 72h. Reactions were stopped by adding 6X protein loading buffer (final concentration of 1X) followed by heat denaturation at 95°C for 10min and analysis by immunoblotting. For cell treatment experimenta,ons, 1 mL of nonprecipitated CHO-LTBP1 CM from the same set of harvest was incubated with 200 nM GzmB (or equivalent volume of PBS) at 37°C for 24 or 48h before being use as culture medium.

#### Purified recombinant human LTBP1

1 μg of rhLTBP1-EGFP was digested with rhGzmB (200 nM, Bon Opus) in 50 nM Tris (Fisher Bioreagents) diluted in PBS, pH 7.4, at 37°C for 24h (final volume of 50 μL). Reactions were stopped by adding 6X protein loading buffer (final concentration of 1X) followed by heat denaturation at 95°C for 10 min and analysis by immunoblotting.

### Cell stimulation and protein extraction

1,000,000 HaCaTs or 500,000 primary dermal human fibroblasts were seeded in 6 well plates (Corning) and cultured for 24h in complete medium before being serum starved for 16 to 24h. For medium transfer experimentations, the supernatant was removed post starvation and cells were cultured in the presence of LTBP1 CM (from the same set of harvest) predigested with 200 nM GzmB for 24 or 48h at 37°C, or with primary human dermal fibroblast CM predigested with 200 nM GzmB for 1h at 37°C. TGF-β1 (from 5 to 10 ng/mL, PeproTech) or GzmB (280 ng or 200 nM, depending on the experimental design and the digestion volume) stimulations were used as both positive and negative controls. After 1h stimulation, cells were washed twice with cold PBS and total proteins were extracted using radioimmunoprecipitation assay (RIPA) extraction buffer containing EDTA-free protease inhibitor and phosphatase inhibitors (Roche) for 20min on ice. After centrifugation at 13,200g for 10min at 4°C, supernatant containing solubilized proteins was stored at -20°C prior to immunoblotting. Protein concentration was determined using the Pierce™ BCA Protein Assay Kit (Sigma-Aldrich).

For TGF-β neutralization experiments, mouse monoclonal IgG anti-TGF-β1,2,3 (MAB1835, Clone 1D11) antibody was purchased from R&D Systems and isotype-matched mouse Kappa Globulin (clone p3.6.2.8.1) used as negative control was purchased from Invitrogen (14-4714-85). In both cases, cells (or LTBP1 CM) were pre-incubated for 10 min at 37°C in the presence of 2.5 μg/mL of either anti pan-TGF-β or isotype-matched antibody before stimulation. For GzmB inhibition experiments, VTI-1002 (viDA Therapeutics, Vancouver, Canada, BC) diluted in DMSO was used at a final concentration of 1 or 2 μM.

### Phase contrast analysis

500,000 CHO-LTBP1 cells were seeded in a 6-well plates for 24h in complete medium before being serum starved overnight. The next day, medium was replaced with fresh FBS free DMEM and cells were treated with GzmB (50 or 100 nM) or an equivalent volume of PBS. After 48h treatment, media was replaced by fresh FBS free medium, and cells were treated another time with the same amount of GzmB or PBS. Cells were cultured for another 5 days in the presence of the protease (total duration of the treatment: 7 days) followed by morphological analysis. Phase contrast pictures were taken using a digital inverted microscope (EVOS FL, Life Technology) at 10X magnification.

### Immunoblotting

20 μg of total proteins extracted from keratinocytes or primary human dermal fibroblasts were analyzed by SDS-PAGE in Tris-Glycine buffer (TG, composed of Tris-base from Fisher Bioreagents and Glycine from Fisher chemical) containing Sodium-Dodecyl-Sulfate (SDS, BIO-RAD) before being transferred onto a Polyvinylidene Fluoride membrane (PVDF 0.2 μm pore size, BIO-RAD) at 0.4 A for 2h in TG buffer containing 10% Ethanol (VWR). Membranes were blocked using 10% (w/v) non-fat dry milk diluted in Tris-buffered saline (TBS, Tris-base and NaCl from Fisher Bioreagents) containing 0.1% (v/v) Tween-20 at room temperature for 1h and incubated overnight at 4°C with primary antibody (orbital shaker). The next day, HRP-conjugated secondary antibody was incubated for 1h at RT and revelation was performed using the SuperSignal™ West Pico PLUS chemiluminescent Substrate (Thermo Scientific). Images were acquired on LI-COR Odyssey Fc system (LI-COR Bio-sciences, Lincoln, NE) and analyzed/quantified using the FIJI software (SciJava). Primary and secondary antibodies dilution are detailed in **Table 2**. For **Supplementary Figure 3C**, 200 ng of recombinant human decorin was used as control and purchased from Abnova.

### Quantification and statistical analysis

Quantification of the number of GzmB^+^, MCT^+^ and CD8^+^ cells in the upper and lower dermis has been performed according to the following methods: each immunobiological section has been pictured at 10 different areas (20X zoom from the 20X magnification) and ranked from one to ten. Then, 5 pictures were randomly selected, and the number of positive cells was counted. The mean was then calculated as the average number of positive cells from these 5 pictures. For decorin quantification, images were deconvoluted on FIJI (SciJava) using the “*deconvolution*” plugin and measured using a 60/240 threshold.

The R software (and its graphical user interface R Commander, T Studio Team, PBC, Boston, MA) and Prism software (GraphPad Software, San Diego, CA, USA) were used for graphical representations of data and statistical analyses. Mean comparison between healthy skin, keloid scars and hypertrophic scars was performed using the Kruskal-Wallis test (non-parametric equivalent of ANOVA) followed by Dunn’s multiple comparison (*vs.* healthy skin). Correlation coefficients r between GzmB and MCT, as well as GzmB and CD8, were determined using the Spearman rank-order (non-parametric equivalent of the Pearson product-moment) correlation coefficient. Comparison of the average number of GzmB^+^/MCT^+^ cells in the upper dermis of LTBP1^+^ and LTBP1^-^ lesions were performed using unpaired Student’s t-test (parametric) after normality (Shapiro-Wilk test) and homoscedasticity (Fisher’s exact test) validation. *p* values < 0.05 were considered as statistically significant and are indicated in the figure legends.

## Data Availability

The original contributions presented in the study are included in the article/**Supplementary Material**. Further inquiries can be directed to the corresponding author.

## Supplementary material

Supplementary Figure 1
**(A)** CD8 immunostaining performed on the upper and lower skin dermis of healthy controls and patients with keloids or hypertrophic scars. Bars, 200 μm. **(B, C)** Quantification of the average number of CD8^+^ T-cells in the upper **(B)** and lower **(C)** dermis of healthy skin controls (n=6, orange), keloids (n=10, pink) or hypertrophic scars (n=10, brown) samples. Results are represented as mean ± SD. **(D, E)** Correlation between the average number of GzmB^+^ and CD8^+^ cells in the upper **(D)** and lower **(E)** dermis of keloids or hypertrophic scars samples (n=20). **p* < 0.05. ns, not significant.

Supplementary Figure 2 Multiplex staining with Tyramide Signal Amplification (TSA) of GzmB (green) and MCT (orange) in the skin dermis of keloids or hypertrophic scars. Scale, 20 μm. Merge showcases overlap of the two channels and co-stained cells appear in yellow. Cell nuclei are stained with DAPI (blue).

Supplementary Figure 3 Immunostaining demonstrating the presence (left panel, 2 out of 10) or absence (right panel, 8 out of 10) of LTBP1 at the dermal epidermal junction of hypertrophic scars (n=10). Scale, 200 μm.

Supplementary Figure 4 Immunostaining demonstrating the presence (left panel, 4 out of 10) or absence (right panel, 6 out of 10) of LTBP1 at the dermal epidermal junction of keloids (n=10). Scale, 200 μm.

Supplementary Figure 5
**(A)** Phase-contrast microscopy performed on Chinese Hamster Ovary (CHO) cells stably transfected with a plasmid encoding LTBP1 and cultured for 7 days under serum deprivation and in presence of PBS or GzmB (50 nM and 100 nM). Arrowheads are pointing at cells undergoing partial EMT and displaying fibroblastic-like features. Bars, 400 μm. **(B)** Western blot analysis showing P-Smad2 and GAPDH levels in HaCaTs cultured in presence of LTBP1 CM for 30 min, 1h, 2h and 3h. **(C)** Western blot analysis showing the presence of LTBP1 (upper panel) and decorin (lower panel) in 1 mL of precipitated LTBP1 CM or 200 ng of recombinant human decorin.

Supplementary Figure 6
**(A)** 1 μg of rhLTBP1 purified by affinity chromatography was incubated with (or without) 280 ng rhGzmB (corresponding to 200 nM in a final digestion volume of 50 μL) for 24h at 37°C and analyzed by western blot probing against LTBP1 (upper panel) and GzmB (lower panel). **(B)** Western blot analysis showing P-Smad2, total Smad2/3 and GAPDH levels in HaCaTs cultured for 1h in presence of TGF-β (3.5 ng/mL) or increasing quantity of purified rhLTBP1. **(C)** Western blot analysis showing P-Smad2, total Smad2/3 and GAPDH levels in HaCaTs cultured for 1h in presence of TGF-β (10 ng/mL), GzmB (280 ng/mL), non-digested rhLTBP1 (1 μg) or rhLTBP1 (1 μg) pre-digested for 24h with 200 nM GzmB (280 ng) and pre-incubated for 10 min with 2 μM VTI-1002. Digestions were performed in a final volume of 50 μL. UT, untreated. ND, non-detectable.

Supplementary Figure 7
**(A, B)** Western blot analysis showing P-p38, total p38 and GAPDH levels **(A)**, as well as P-p44/42, total p44/42, and GAPDH **(B)** levels in HaCaTs cultured for 1h as described in (**Figure 7B**). **(C, D)** Western blot analysis showing P-p38, total p38 and GAPDH levels **(C)**, as well as P-p44/42, total p44/42, and GAPDH **(D)** levels in HaCaTs cultured for 1h as described in (**Figure 7B**) in presence (or not) of an anti-pan-TGF-β inhibitor. UT, untreated. ND, non-detectable.

Supplementary Figure 8 Western blot analysis showing P-Smad2, total Smad2/3 and GAPDH levels in primary dermal human fibroblasts cultured for 1h in presence of TGF-β (5 ng/mL), GzmB (280 ng/mL), non-digested rhLTBP1 (1 μg) or rhLTBP1 (1 μg) pre-digested for 24h with 200 nM GzmB (280 ng). Digestions were performed in a final volume of 50 μL. UT, untreated. ND, non-detectable.
