# Supplementary material for: Potential implications of granzyme B in keloids and hypertrophic scars through extracellular matrix remodeling and latent TGF-β activation
Source: Front Immunol. 2025 Jan 16;15:1484462. doi: 10.3389/fimmu.2024.1484462 (PMC11779620; doi:10.3389/fimmu.2024.1484462)

Supplementary Figure 1

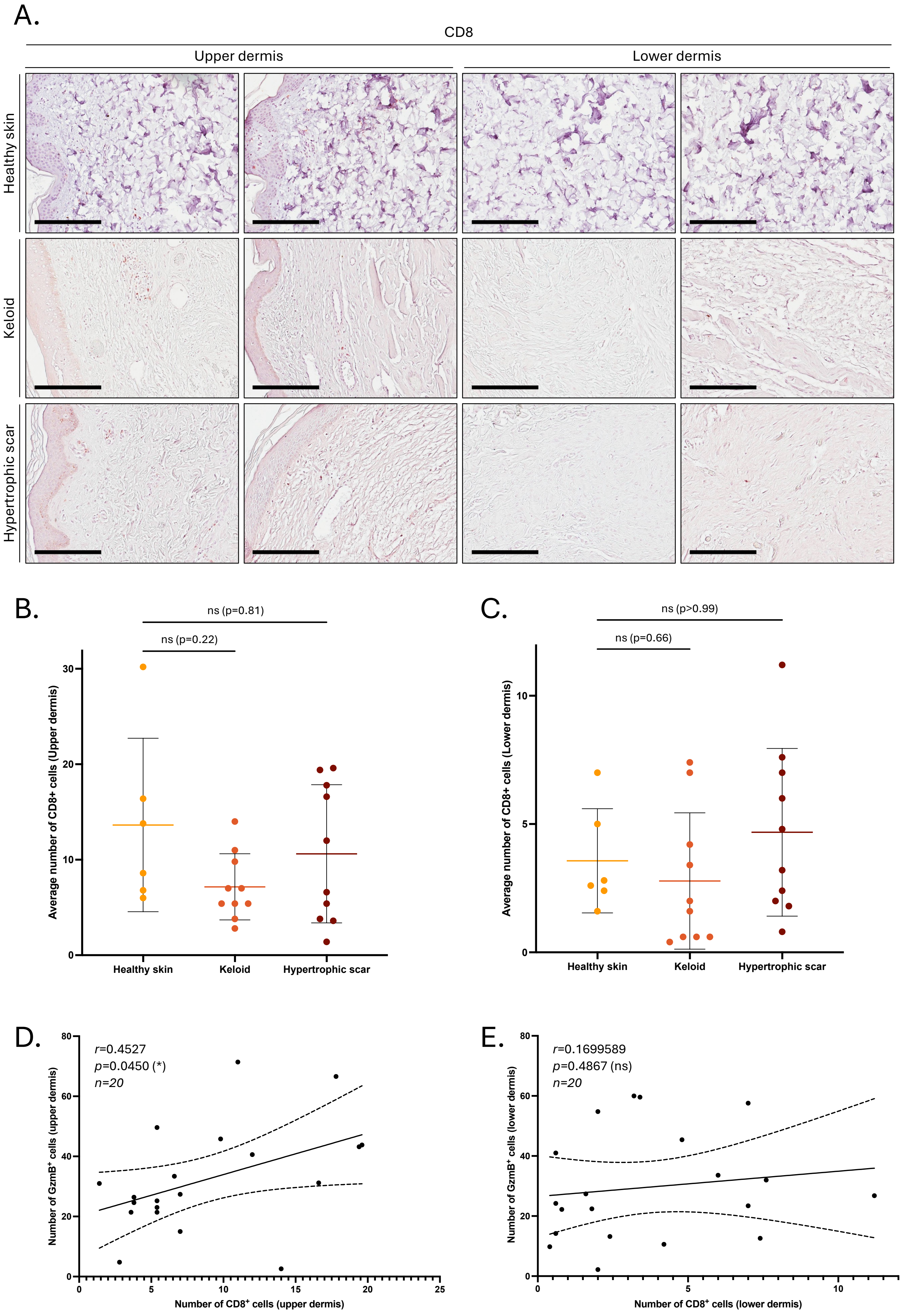

Supplementary Figure 2

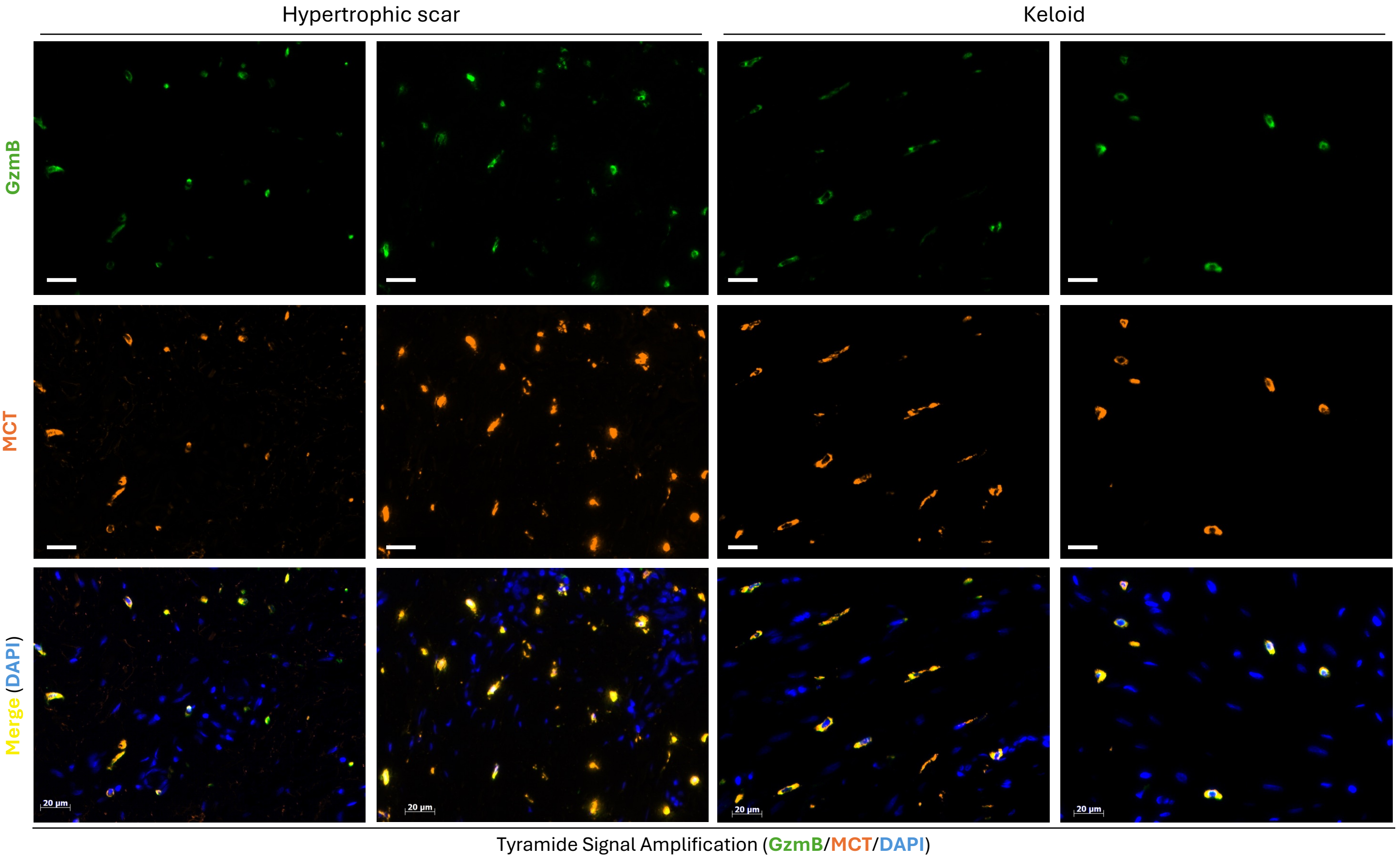

Supplementary Figure 3

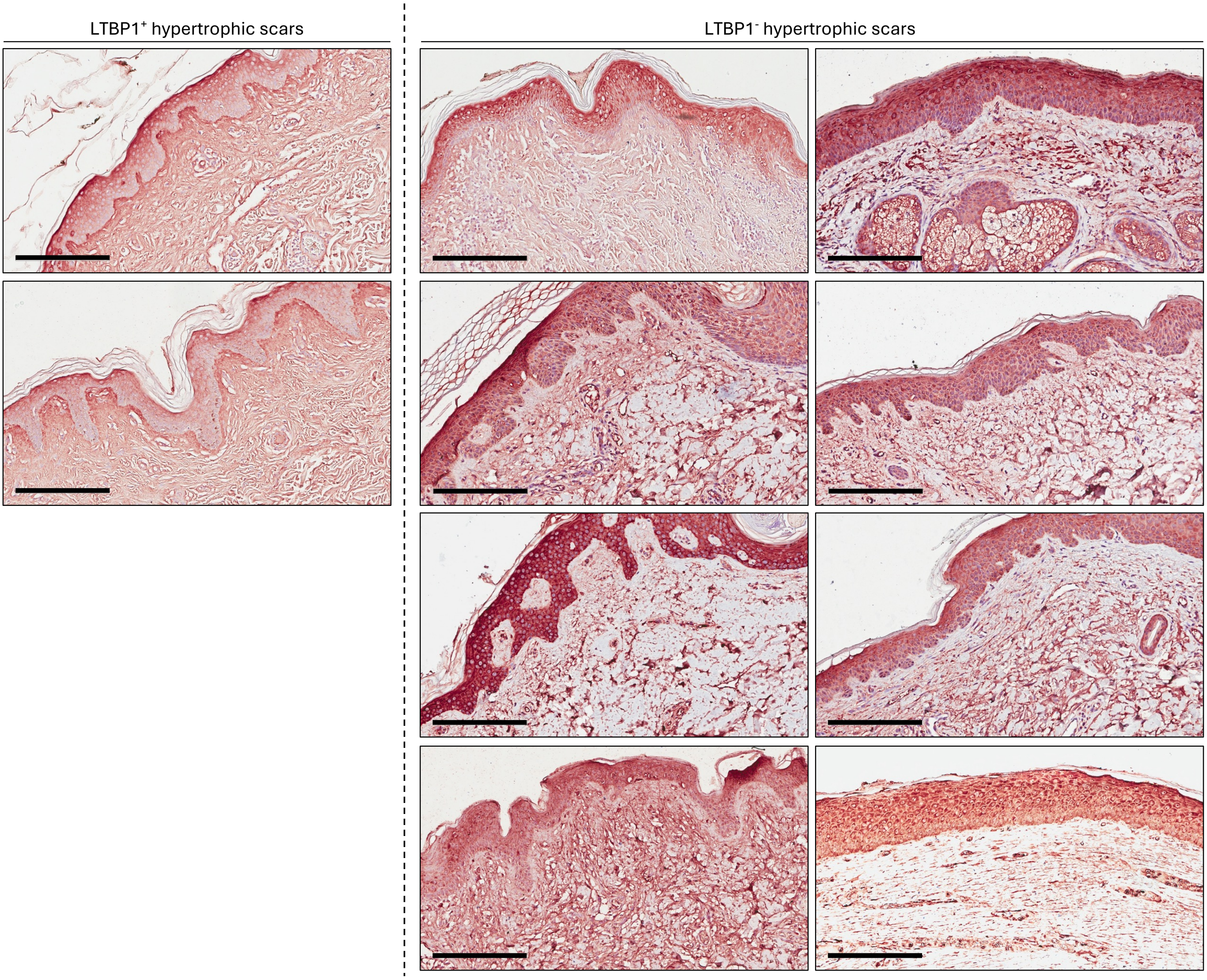

Supplementary Figure 4

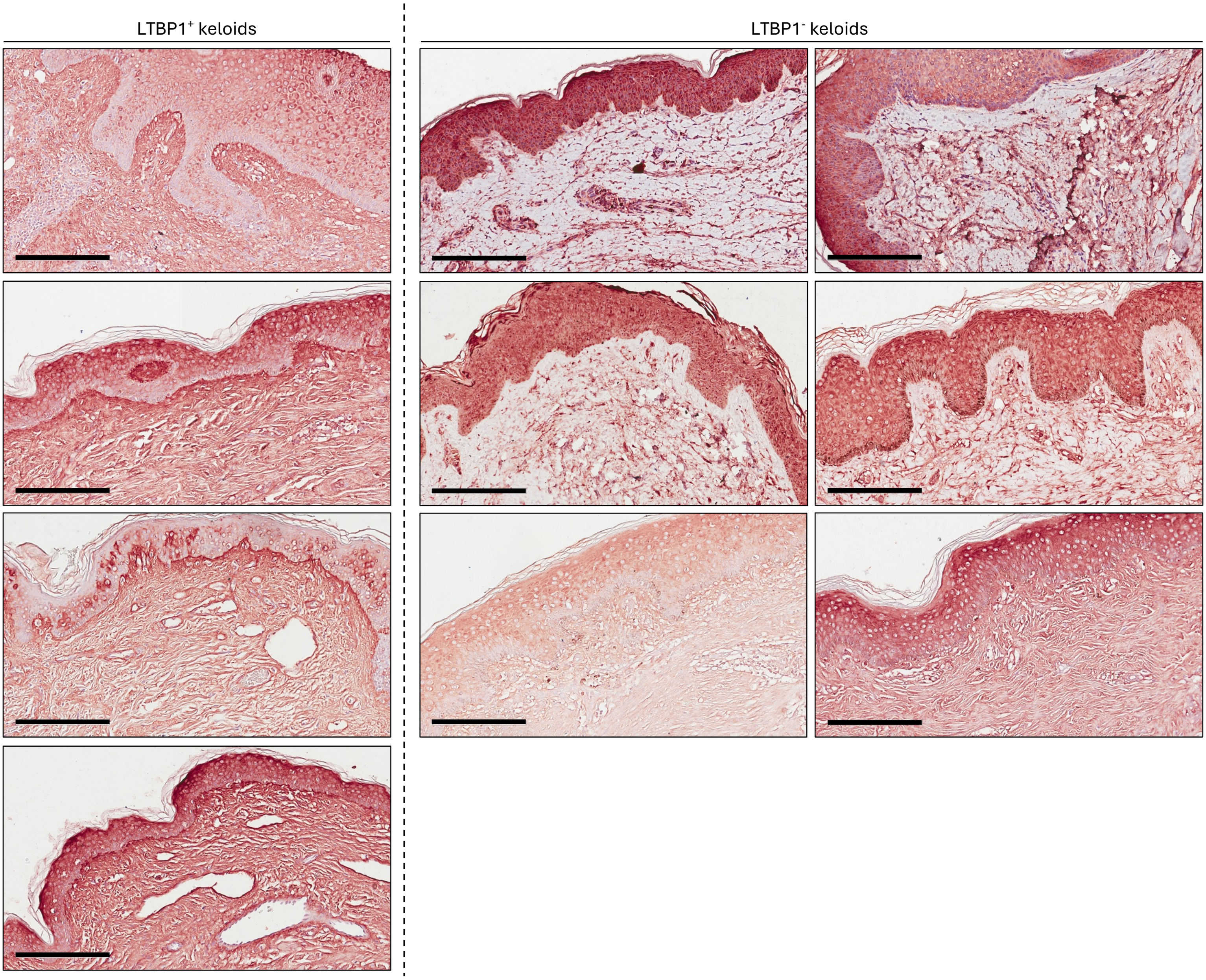

Supplementary Figure 5

A.

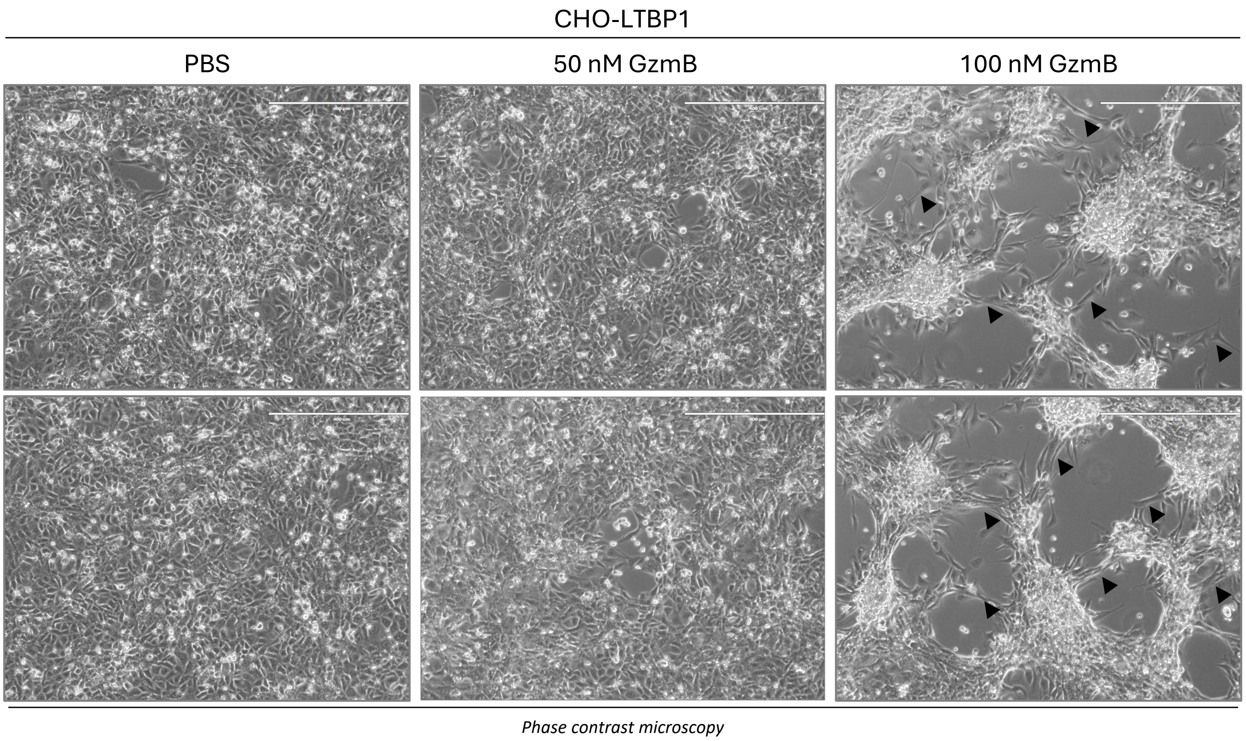

B. *Keratinocytes (HaCaT)*

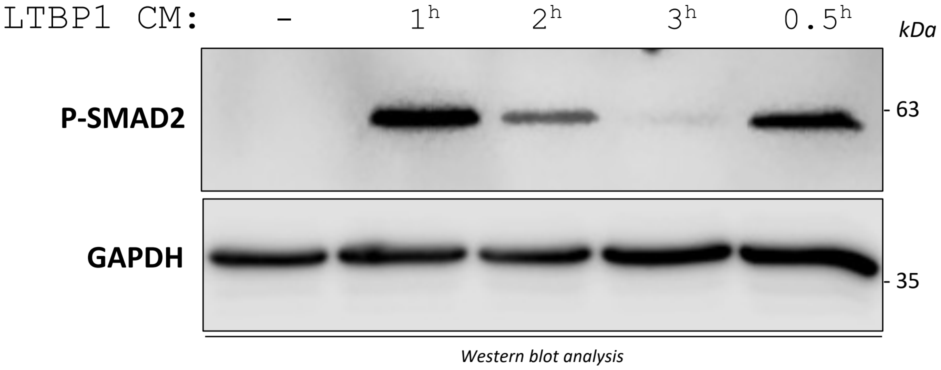

C.

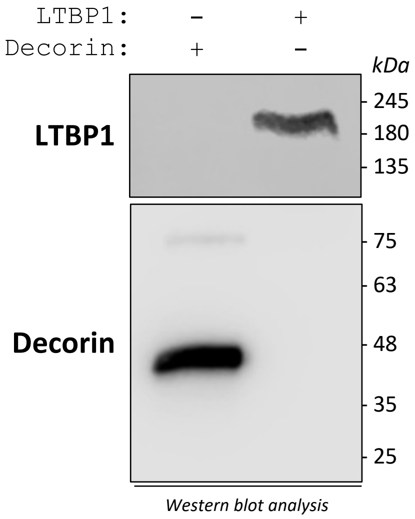

Supplementary Figure 6

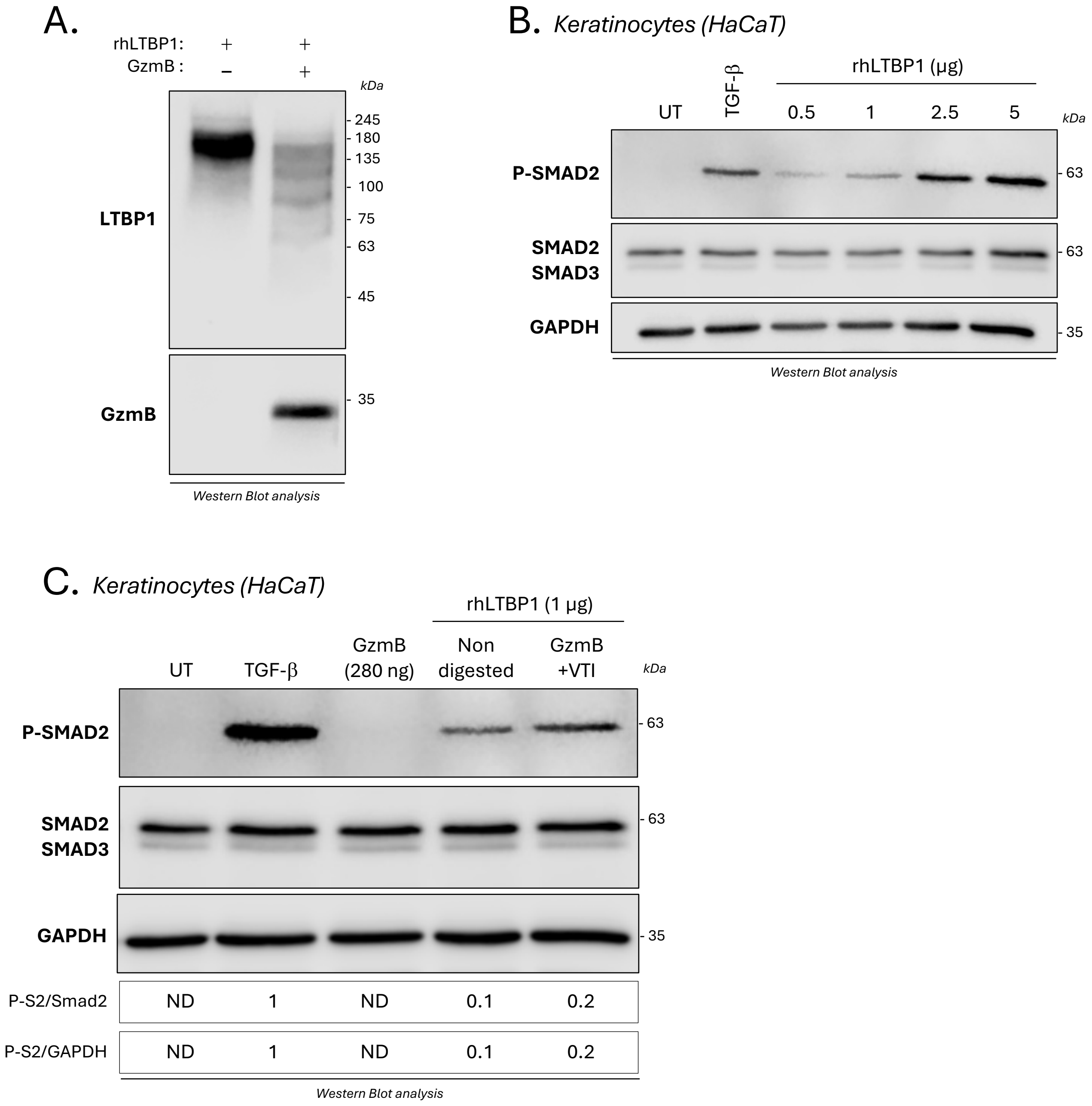

Supplementary Figure 7

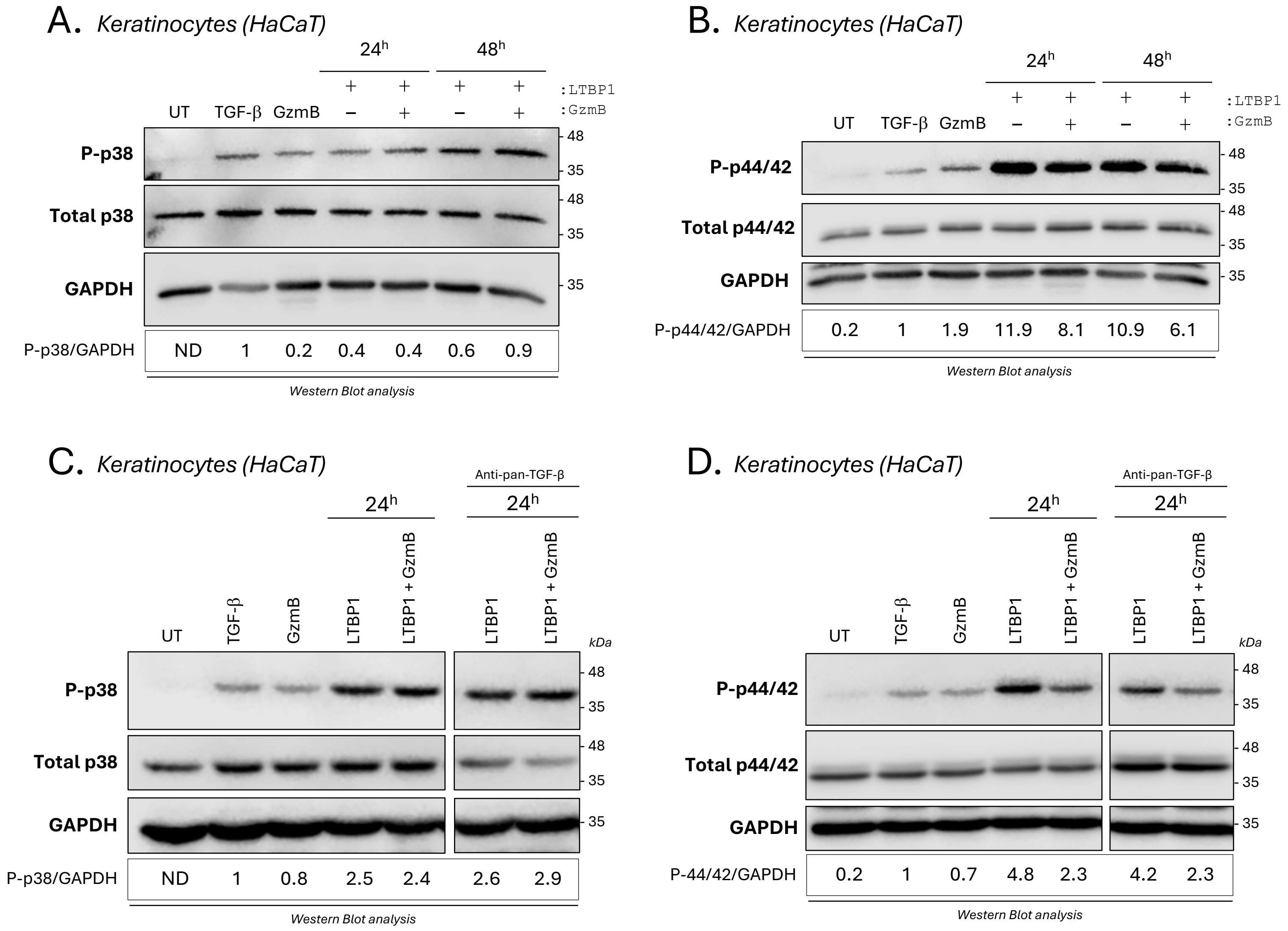

Supplementary Figure 8

Primary human dermal fibroblasts

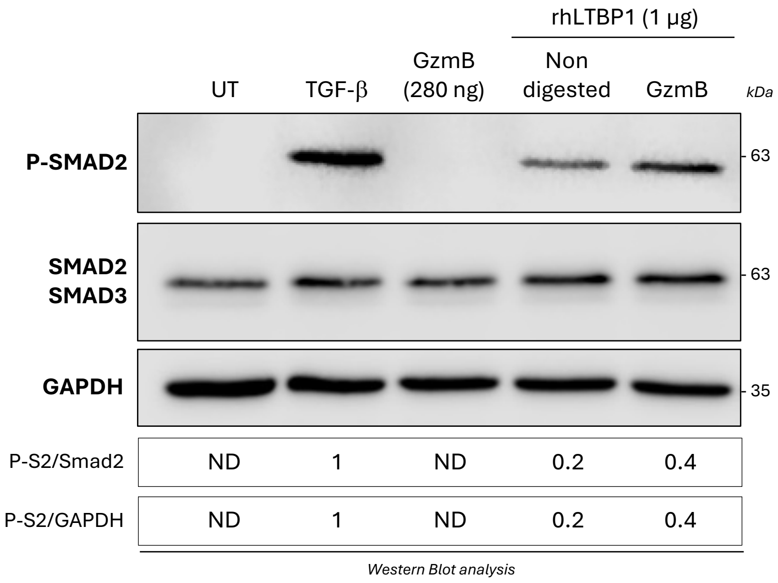

Supplement: Supplementary Figure 1 — (A) CD8 immunostaining performed on the upper and lower skin dermis of healthy controls and patients with keloids or hypertrophic scars. Bars, 200 μm. (B, C) Quantification of the average number of CD8+ T-cells in the upper (B) and lower (C) dermis of healthy skin controls (n=6, orange), keloids (n=10, pink) or hypertrophic scars (n=10, brown) samples. Results are represented as mean ± SD. (D, E) Correlation between the average number of GzmB+ and CD8+ cells in the upper (D) and lower (E) dermis of keloids or hypertrophic scars samples (n=20). *p < 0.05. ns, not significant. [file DataSheet1.pdf]
